# Gene Expression Analysis in the Thalamus and Cerebrum of Horses Experimentally Infected with West Nile Virus

**DOI:** 10.1371/journal.pone.0024371

**Published:** 2011-10-04

**Authors:** Melissa A. Bourgeois, Nancy D. Denslow, Kathy S. Seino, David S. Barber, Maureen T. Long

**Affiliations:** 1 Department of Infectious Diseases and Pathology, University of Florida College of Veterinary Medicine, Gainesville, Florida, United States of America; 2 Department of Physiological Science, University of Florida College of Veterinary Medicine, Gainesville, Florida, United States of America; 3 Department of Veterinary Clinical Sciences, Washington State University College of Veterinary Medicine, Pullman, Washington, United States of America; Virginia Polytechnic Institute and State University, United States of America

## Abstract

Gene expression associated with West Nile virus (WNV) infection was profiled in the central nervous system of horses. Pyrosequencing and library annotation was performed on pooled RNA from the CNS and lymphoid tissues on horses experimentally infected with WNV (vaccinated and naïve) and non-exposed controls. These sequences were used to create a custom microarray enriched for neurological and immunological sequences to quantitate gene expression in the thalamus and cerebrum of three experimentally infected groups of horses (naïve/WNV exposed, vaccinated/WNV exposed, and normal).

From the sequenced transcriptome, 41,040 sequences were identified by alignment against five databases. 31,357 good sequence hits (e<10^−4^) were obtained with 3.1% of the sequences novel to the equine genome project. Sequences were compared to human expressed sequence tag database, with 31,473 equine sequences aligning to human sequences (69.27% contigs, 78.13% seed contigs, 80.17% singlets). This indicated a high degree of sequence homology between human and equine transcriptome (average identity 90.17%).

Significant differences (p<0.05) in gene expression were seen due to virus exposure (9,020), survival (7,395), and location (7,649). Pathways analysis revealed many genes that mapped to neurological and immunological categories. Involvement of both innate and adaptive components of immunity was seen, with higher levels of expression correlating with survival. This was highlighted by increased expression of suppressor of cytokine signaling 3 in horses exposed to WNV which functions to suppress innate immunity. Pentraxin 3 was most increased in expression for all horses exposed to WNV.

Neurological pathways that demonstrated the greatest changes in gene expression included neurotransmitter and signaling pathways. Decreased expression of transcripts in both the glutamate and dopamine signaling pathways was seen in horses exposed to WNV, providing evidence of possible glutamate excitotoxicity and clinical signs associated with decreased dopamine. Many transcripts mapped to non-infectious neurological disease functions, including mental disorders and degenerative neuropathies.

## Introduction

West Nile virus (WNV) is one of the leading causes of arboviral encephalitis in the United States for both horses and humans. The virus can be devastating in its ability to cause long term neurological deficits and even death. Since WNV was introduced into the United States in 1999, 25,791 clinical cases of disease have been confirmed in horse [1]. Similarly, in humans, 30,625 confirmed cases of clinical disease have been reported with 1,202 deaths [2]. The arbovirus season of 2010, ten years after introduction, was notably active with WNV activity detected in all continental states. Human cases were detected in 42 states with 89 infections reported in New York and 105 infections detected in the opposite extreme geographically in Arizona. Most of the cases reported were of the neuroinvasive form and 45 deaths occurred. The US blood supply continues to be at risk with 144 detections of viremic blood obtained from donors in 2010 alone [2].

Characterization of WNV infection provides an opportunity to enhance our understanding of the pathophysiology and immunopathology of viral encephalitis in general. This is particularly important in regards to host-pathogen interactions during infection. During neuroinvasive disease, WNV mainly infects the neurons of the midbrain, hindbrain, and spinal cord, with limited infection of glial cells. Direct viral infection of CNS cells followed by induction of the inflammatory response allows analysis of the virus from systemic infection, to neurological spread, through stages of innate and adaptive immunity for the purposes of biomarker and interventional strategies. Investigation of one of the two most commonly affected natural mammalian hosts allows modeling of this disease under both experimental and field settings.

WNV infection in natural mammalian hosts is characterized clinicopathologically by an increasing number of lesions progressing from the diencephalon through the hindbrain and down through the spinal cord. Congestion of the meninges and hemorrhagic foci may be seen. Comparatively, mice develop disease that is primarily focused in a cerebral and cerebellar localization of virus. Histopathologically, inflammatory lesions characterized by layer of monocellular perivascular cuffing and gliosis are present in horses, humans, and mice, while necrosis is the primary lesion in hamsters [3], [4]. This pathology is likely a result of many complex factors, as there is increasing evidence that the host response to viral infection is one of the main causes of pathology and survival during WNV infection in the CNS [5], [6]. In humans and horses, long term recovery responses are variable, with evidence of moderate to severe congnitive, emotional, and motor deficits in humans, and behavioral and motor deficits in horses [7]. Thus there is a gap in our understanding of the interactions between various components of the natural host response to infection, and how these interactions can lead to pathology, long term deficits, or full recovery. Given the similarities between disease in horses and humans, equine tissue analysis offers an unparalleled opportunity to profile gene expression and pathway interactions between pathogens and the CNS.

Although there are many new methods for profiling gene expression, there is limited development of *de novo* deep sequencing strategies due to limited financial resources and species specific bioinformatics in veterinary research fields. In terms of adaptability and computational resources, microarrays allow rapid acquisition of tissue specific expression data for many non-model species. Microarrays have facilitated the study of the Flaviviridae in multiple applications including detection of variants of Dengue virus (DV) in human samples [8], differentiation between different flaviviral and other viral infections [9], [10], and mutations in the structural regions of the WNV genome [11]. Microarrays have been used to analyze gene expression at both the cell culture and organism level for DV, JEV, and yellow fever virus (YFV) infection [12], [13], [14], [15], [16], [17]. Although several species specific arrays exist, there is limited development of tissue specific arrays in veterinary medicine. The microarray currently developed in the horse is based on global gene expression in which multiple tissues of the equine transcriptome were sequenced [18]. While a valuable tool, only cerebrum, cerebellum and spinal cord were utilized in this work to generate transcriptome data.

Two studies have used microarrays to examine gene expression changes in response to WNV infection. In one, human glioblastoma cell culture transcriptional responses to WNV were analyzed, and 23 genes involved in neurodegenerative disorders were shown to be changed in expression [19]. In the other, a microarray was used to analyze whole organism gene expression response to WNV strains of different neurovirulence in a mouse model [20]. Genes involved in immunological, neurological, and apoptotic functions were differentially regulated (with forty-seven genes shown to be upregulated in highly neuroinvasive strains).

No studies are available that profile gene expression in the CNS of animals infected with WNV that are considered natural, susceptible hosts. These data could provide detailed information on the host response to infection and on a pathogen's specific manipulation of the host response, and will also allow more efficient analysis of essential pathways in model species such as mouse and hamster. This report provides sequencing data from the equine brain transcriptome and lymphoid system from naïve horses experimentally infected with WNV, vaccinated horses experimentally infected with WNV, and negative controls. Also described is the construction of a custom, validated equine high density microarray, in which pathways of the CNS and immune system were enriched. The microarray was used to profile gene expression changes in the thalamus and cerebrum of naïve and vaccinated horses during experimental WNV infection (and negative controls) and common gene pathways were identified. These data were used to detect differences in gene expression associated with exposure to WNV, survival from WNV infection, and location of WNV infection in the brain.

## Materials and Methods

### Use of Animals

All animals used in this study were used in strict compliance with the Guide for the Care and Use of Laboratory Animals. The protocols for the use of animals were approved by the University of Florida Institutional Animal Care and Use Committee (IACUC protocols F077, F093, D163). All efforts were made to minimize pain and suffering.

### Sample Selection

All tissues used in this study for library creation and array analysis were derived from horses used in an experimental intrathecal challenge model wherein naïve horses developed grave West Nile (WN) encephalitis (100% nonsurvivorship) and all vaccinated horses did not develop clinical disease (100% survivorship). Samples for pyrosequencing and array analysis consisted of 1) naïve horses infected intrathecally with 1×10^5^ WNV/NY99/crow, 2) non naïve horses vaccinated utilizing a modified live attenuated Yellow Fever (YF) chimera vaccine for protection against WNV (Prevenile, Intervet-Schering-Plough) and infected intrathecally with 1×10^5^ WNV/NY99/crow , and 3) horses that were not infected or vaccinated. Experimental infection and vaccination of horses occurred according to previously published data [4], [21], [22]. Horses from groups 1 and 2 were euthanized (University of Florida IACUC protocols #F077, #F093, #D163) if demonstrating clinical signs or at the end of the study (day 21) if not demonstrating clinical signs. Horses from group 3 were normal healthy horses, not infected with WNV and were euthanized due to other causes (4 of these horses were less than 4 years of age, while 2 were over the age of 9). These causes included lameness (angular limb deformities, chronic lameness) and humane reasons as surrender by owner. None of the horses were ill or demonstrating neurological signs at the time of euthanasia (all horses underwent neurological exams). All horses were necropsied immediately upon euthanasia. Tissues were snap frozen in dry ice and ethanol and stored at −80°C until RNA extraction was performed. Eight tissues were collected from each horse and included cerebrum, cerebellum, thalamus, midbrain (rostral and caudal colliculus, tectum, and tegmentum), hindbrain (pons and medulla oblongata), cervical spinal cord, lumbar spinal cord, and spleen.

A pooled tissue strategy was used to create a library that was normalized for rare sequences that might not have been annotated in the EqCab2 database, and that allowed for longer reads than that accorded by directly sequencing short reads from each individual horse. A cDNA library was constructed from a pool of tissues mentioned above from six different horses including two gravely ill naïve horses infected with WNV; two horses vaccinated, infected, and recovering from WNV(vaccinated, intrathecally infected with WNV, and survived without showing clinical signs that warranted euthanasia according to IACUC protocols); and two control horses that were not infected or vaccinated. Eight tissues taken from each horse were pooled together for a final total of 5 samples from individual horses, each containing the eight tissues. Brain tissues used to create the cDNA for dye labeling on the array were obtained from the archived tissues of three groups of six horses each (total of 18 individuals) mentioned above. Tissues used in the array included cerebrum and thalamus (one section from each horse for a total of 36 samples).

Three analyses were established to test the hypothesis that there are gene pathways whose expression changes in a significant and consistent manner due to WNV as a result of exposure status, survival/immune status, and CNS location (Table 1). With respect to the experimental analyses, three subhypotheses were generated to analyze if there was a difference in gene expression between the nonvaccinated/exposed and untreated horses (exposure), the nonvaccinated/exposed and vaccinated/exposed horses (survival), and the nonvaccinated cerebrum and nonvaccinated thalamus (location). In particular, the “survivors” represent the gene expression status of those animals that recover (vaccinated, intrathecally infected with WNV, and survived without showing clinical signs that warranted euthanasia according to IACUC protocols) from grave WN encephalitis through induction of vaccine mediated immunity, and the “non-survivors” represent the gene expression status of naive animals undergoing grave encephalitis.

**Table 1:** Samples and analyses for the array.

Analysis	Samples	Tissue type	WNV Challenge
Exposure Status	Not vaccinated- 6 horses	Thalamus	Y
	Untreated- 6 horses	Thalamus	N
Survival/Immune Status	Not vaccinated- 6 horses	Thalamus	Y
	Vaccinated- 6 horses	Thalamus	Y
CNS Location	Not vaccinated- 6 horses	Thalamus	Y
	Not vaccinated- 6 horses	Cerebrum	Y

A total of 12 tissues were compared for each of the analyses/questions asked. The questions included determining if there was a difference in gene expression due to exposure to WNV, recovery from WNV infection, and location in the brain.

### Clinical Signs and Histopathological Grading

Neurological exams and clinical scoring were performed on horses in all groups weekly after vaccination and daily after infection for the horses exposed to WNV as according to previously published data [4], [21], [22]. Neurological exams and clinical scoring were also performed upon receipt for the normal control horses. Briefly, horses were evaluated for changes in mentation, paresis, ataxia, and muscle fasciculations. Clinical signs and temperature were recorded from day −1 to day 21 for the horses exposed to WNV (vaccinates and non-vaccinates). Non-vaccinated horses and vaccinated horses exposed to WNV were euthanized for humane reasons according to University of Florida IACUC protocols #F077, #F093, #D163 if demonstrating severe clinical signs or neurological disease, or at the end of the study (day 21). Normal control horses were euthanized according to University of Florida IACUC protocols #F077, #F093, #D163 upon receipt.

Blood was drawn from each horse exposed to WNV on days 1–14 post-infection. A PFU assay was performed on the heparinized blood and cells observed for cytopathic effect to determine levels of viremia. Histopathological grading was performed on sections from the thalamus and cerebrum to investigate for the presence of viral encephalitis (gliosis and perivascular cuffing). Scoring was performed by two blinded and independent pathologists according to previously published data [22]. Lesions were quantified in the pons, medulla, cervical cord and lumbar cord. Briefly cross sections of these areas were examined for the presence of gliosis and perivascular cuffing. One section each was evaluated for the pons and medulla. Two sections were evaluated for each area of the spinal cord. Total numbers of glial nodules were counted in each section. If more than one section was evaluated the counts for these sections were averaged. For pervascular cuffs, 3 areas were examined in each section and 10 vessels were counted in each area. The number of vessels that contained inflammatory cells was divided by the number 10. Each area per section was averaged.

### Creation and Annotation of a Normalized CNS/Lymphoid Tissue cDNA Library

The RNA isolated from the tissue samples from each horse were pooled to create 5 samples total (eight tissues taken from each horse were pooled together for a final total of 5 samples from individual horses, each containing the eight tissues). The quality of the RNA was checked using spectrophotometric technology (ND-1000, Nanodrop Technologies, Wilmington, DE) and a microfluidics platform (Agilent 2100 Bio-analyzer, Santa Clara, CA). The RNA was converted to full-length, double-stranded cDNA using commercial cDNA synthesis kits (SMART PCR cDNA synthesis kit, Advantage® 2 PCR kit, and PowerScript Reverse Transcriptase, Clontech, Mountainview, CA). Concentration and purity of the cDNA were assessed (Methods S1) and the purified cDNA sample was normalized using a commercial kit encorporating an endonuclease strategy ( cDNA Normalization Trimmer Kit, Evrogen, Moscow, Russia) according to manufacturers' instructions.

An initial titration run was performed to ensure transcript normalization and then two full pyrosequencing runs (Gene Sequence 20, 454 Life Sciences, Branford, CT) were performed on the cDNA pool. The pyrosequencing data was directly captured by the accompanying manufacturer's software (Peak Height Determination Software , PyrosequencingAB), and was initially assembled using short-read software assemblers (Ensembl, European Bioinformatics Institute/Wellcome Trust Sanger Institute; Newbler, 454 Life Sciences) to identify singlets and contiguous, non-redundant sequences. For generation of a larger set of nonredundant sequences (contigs), additional sequence cleaning, sequence clustering, and assembly was performed using software that aligns and detects alternative splice forms (PTA; Paracel Transcript Assembler, Paracell Inc, Pasadena, CA). Annotation of these sequences was performed with the Basic Local Alignment Search Tool analysis (BLAST) quest algorithm for storage, management, and analysis of EST sequences (NCBI). This consisted of a homology search using the BLASTX and BLASTN against the NCBI databases (NT Nucleotide Database, NR Protein Database), and equine databases (Horse draft genome database EqCab2, EqCab2 predicted genes, and EqCab2 gene scan). Only genes that were positively identified by BLAST with expected values (e-values) below 10^−4^ were used to compile the results.

BLAST results were further cleaned and stored in BlastQuest (Interdisciplinary Center for Biotechnology Research, UF, Gainesville, FL) to facilitate management of BLAST results and the Rare Ontology Consortium (GO) term browsing. Software called Assembly Filter (ICBR, UF, Gainesville, FL) was used to query the top 100 BLAST hits for each contig against the NCBI Gene database. This provided annotation information, gene function, and metabolic pathway associations based on GenMAPP and KEGG pathway database maps. The GO terms and pathway information associated with the lowest e-value and consistent with the NCBI databases were assigned to the query assembly process. Contigs with the highest agreement were maintained and the least similar sequences were eliminated. Sequence orientations were determined by software instruments, AssemblyFilter and ESTscan (EMBnet, Switzerland). Further analysis of sequences that were missed by the equine genome database was then performed to determine similarities in function by grouping like GO categories. Sequences were analyzed for species composition between the NT and NR NCBI databases. The complete sequenced transcriptome was run against the human expressed sequence tag database (Fisher cluster, UF, Gainesville, FL) to determine sequence homology between the human and horse. An e-value of <10^−4^ was set and only one match per sequence generated.

### Microarray Design and Analysis

Probe sequences were based on the sequenced and annotated cDNA library. Probes consisted of oligonucleotides (60-mer) fabricated by a patented algorithm (Agilent Technologies, Santa Clara, CA) based on the annotated equine brain library and a 44,000 gene array (Agilent Technologies, Santa Clara, CA). Preference was given to probes with the greatest length, greatest abundance, and lowest e-value (<10^−4^) within a cluster (set of similar sequences). All designed probes were included with one replicate each in 1) annotated, 2) annotated minus orientation, 3) unannotated, and 4) recovered genome categories (Table S1). Several probes consisting of neurological, immunological, and cell death gene ontology categories were considered to be of particular importance and replicates were included on the array. Uniquely designed probes (250) designed by the manufacturer (Agilent) were also included as technological controls on the arrays.

Total RNA was extracted and quality assessed from a total of 36 tissues (cerebrum and thalamus from 6 horses in 3 different analyses) using the protocol outlined in Methods S1. Dye-labeled cDNA was created using Cy3 dye (One-Color Microarray-Based Gene Expression Analysis kit, Agilent Technologies) according to the manufacturer's protocols (See Methods S1) except for minor protocol changes dictated by the CNS tissue. Hybridization to the microarrays was performed according to the manufacturer's protocol (Agilent Technologies) outlined in Methods S1. All data was submitted to the GEO microarray database according to MIAME standards. The series record number GSE30347 was assigned.

### Normalization and Statistical Analysis

JMP Genomics version 4.0 (S.A.S. Institute, Cary, NC) was used to analyze the data. All files were transformed (log_2_) and normalized using Loess normalization techniques. Normalization was verified using distribution analysis consisting of box plots, correlation heat maps, and overlayed kernel density elements, and principal component analysis consisting of 2D, 3D, and scree plots. A two-way analysis of variance (ANOVA) was performed (location and treatment were independent variables) and possible interactions between location (cerebrum and thalamus) and treatment (vaccinated, not vaccinated, normal) were tested in the analysis addressing location, exposure, and survival (p<0.05). Only thalamus was compared between the two analyses addressing exposure and survival due to differences in gene expression between the cerebrum and thalamus seen in the normal, non-exposed horses. The degree of fold-change (relative fluorescent intensity) was analyzed for all differentially regulated genes. Variability was estimated in the software via linear regression and Pearson correlation coeffiecient and the R square and residual variance tables were generated for each array. A significant genes lists was generated and a hierarchical clustering was performed.

### Gene Ontology Enrichment

Probes for the analyses of location of the brain, exposure status of the horse, and survival or recovery status of the horse that matched to the gene ontology categories of biological process, cellular component, and molecular function were identified. Gene ontology categories (as derived from the original annotation of the cDNA expression library, Fisher Cluster, University of Florida) that involved the neurological system, immunological system, apoptosis/cell death, and transcription/translation were targeted. The three analyses were analyzed based on the number of significantly different genes that grouped into these GO categories.

### Pathway Modeling

Genes that showed significant up- or down-regulation (p<0.05) with fold changes >+1 or <−1 for all three statistical analyses were extracted and databased for modeling into ontological networks (Ingenuity Pathways Analysis software, Ingenuity Systems, Redwood, CA). Network modeling to determine interactions between significant genes, canonical pathways analysis to determine genes involved in known pathways, and disease/physiological function/location annotation was performed on genes with fold changes >1 and <1 and on significant genes. As part of this computational format, Fisher's exact test was used and both the number of transcripts and p-values considered in ranking of pathways, networks, and functions. This process was performed on all significant genes as well as on the gene ontology enriched datasets.

### Microarray Validation

For the purposes of the initial validation of the utility of this microarray, several highly significant genes (six) were selected to 1) verify the accuracy of the probe hybridization, and 2) verify the accuracy of the relative expression values detected by the probe. To verify the relative expression values, only transcripts that were significantly upregulated or downregulated (p<0.05, fold change >2, <−2) in the exposure analysis were picked for analysis. A total of six transcripts were targeted to be used as primer sets in the validation experiment and included 2′5′ oligoadenylate synthetase (2,5 OAS), complement component 1 (CC1), TNFα receptor ligand (TNFR), interleukin-6 (IL-6) , DEAD Box 60 (DB60), and defensin β4 ( DB4), with β -actin (ACT) as the endogenous control outlined in Methods S1. Two sets of primers were designed using primer design software (ABI Primer Express version 3.0, Applied Biosystems). Conventional PCR was performed using a proprietary master mix (Readymix Taq PCR Mastermix with MgCl_2_, Sigma-Aldrich, St.Louis, MO). The reactions were resolved utilizing a 0.9% agarose gel and imaged under standard UV conditions. If a band(s) was visualized, the samples were submitted to the UF Interdisciplinary Centers for Biotechnology Research for Sanger sequencing. Sequencing results were checked against expected gene sequences. Once the correct sequence was validated, amplified samples were run under the thermocycling conditions listed above using a second set of nested primers. The presence of a band of the correct length was verified on a 0.9% agarose gel. Relative quantitation analysis was performed using the proprietary software for calculation of the comparative Ct method (Applied Biosystems software for the 7500 Fast machine) wherein 2^−ΔΔCt^ is used for the comparison of relative quantitation between the thalamus of vaccinated/exposed horses and nonvaccinated/exposed horses. To verify the accuracy of the probe hybridizations, the probe sequences were BLASTed against the equine genome (Fisher Cluster, UF ICBR, Gainesville, FL). Only sequences with e-values<10−4 were generated. Sequences were checked for percent identity and sequence alignment.

## Results

### Annotation and Analysis of the Sequenced Equine CNS Transcriptome

#### Library Sequencing and Annotation

The quality and purity of the RNA samples (average RIN 7.1–7.6) and cDNA libraries can be seen in Table S2. Successful normalization was confirmed via visualization of the bands on a gel and a titration sequencing run. In total, 308 contigs were identified with an average length of 208.1 base pairs. Only 4 large contigs were identified, confirming that normalization was effective.

After linkers and repetitive sequences were removed, 514,462 sequences were assembled from a total of 49,857,586 bases using Newbler Assembly filter software. Fully assembled reads, partially assembled reads, singletons, repeats, outliers and contigs were identified. A total of 16,895 contigs were identified (1,902 large contigs) (Table S3). The sequences obtained from the three assembly analyses included 16,895 contigs obtained from the Newbler assembly, 443,584 unannotated sequence sets from the 454 sequencing run, and 22,748 sequences from Ensembl. These sequences were databased and assembled using PTA for a total of 188,885 final sequences (Table 2). Clusters were determined (overlap 100 base-pair minimum) and 61,499 sequences matching with ‘seed’ sequences were clustered. Sequences that were not identified as ‘seed’ sequences (127,386) were clustered in three steps via partitioning, pairwise comparison, and clustering.

**Table 2:** Data from 454 Sequencing Runs Paracel Transcript Assembler.

Classification	Number of Sequences
No. of current input sequences	483,227
No. of sequences removed during cleanup	294,342 (609 were EquCabv2 genes)
No. of sequences kept after cleanup	188,885 (22,139 were EquCabv2 genes)
No. of sequences in seed clusters	61,499
No. of sequences pairwise compared	127,386
No. of singlets after pairwise compared	75,413
No. of problem sequences	54
No. sequences in clusters	51,919
No. of seed clusters	21,421
No. of clusters	11,634
Largest cluster	cl.007 (2,998)
2^nd^ largest cluster	cl.015 (2,721)
Largest seed cluster	sd.17584 (112)
2^nd^ largest seed cluster	sd.3613 (105)
No. final assemblies	134,844
No. of cluster.contigs	11,621
No. of cluster singlets	2,098 (4 genes+19 newbler contig+2,075 reads)
No. of seed cluster.contigs	8,058
No. of seed cluster singlets	37,654 (*21,021 genes+29 newbler contig+2,498 reads)
No. of PTA contigs	19,679 (11,621+8,058)
No. of singlet	75,413 (28 genes+260 newbler contig+75,125 reads)
No. of contigs	19,987 ( = 11,621+8,058+19+29+260)
No. of genes	21,053 ( = 4+21,021+28)

Combining the results from Newbler and PTA analyses and utilizing known sequences from the equine genome databases, 19,987 contigs and 21,053 genes were assembled. Unassembled sequences were not considered for further analysis. In total, 41,040 sequences were used in the BLAST analysis [19,679 PTA contigs ( = 11,621 non-seeded contigs+8,058 seeded contigs), 308 newbler contigs and 21,053 unassembled EquCabv2 genes]. These sequences have been submitted to GenBank (NIH) for public access (study # SRP000619).

#### Library Analysis

A BLAST search was run against five separate databases (e-value 10^−4^ ) and 31,357 good sequence hits were obtained (Table 3). Approximately 73.7% of the sequences identified in this project were matched in the equine chromosome database and at least one of the equine predicted genes databases, while 23.1% of the sequences recognized in this project were only identified in the equine chromosome database. Completely novel genes yet to be identified by the equine genome project were represented by 3.1% (1,278) of the sequences identified. The average HspScores and bit scores for all equine databases indicated a high degree of alignment of the sequences with those in the equine databases. All sequences demonstrated significant length for matching. In addition, the majority of sequences for all databases demonstrated positive identity greater than 95% (Table S4).

**Table 3:** Summary of BLAST Results for Five Separate Databases.

Count	%	NR/NT	Equ-Cab2 Chr.	Equ-Cab2 Predicted Genes	Equ-Cab2 GeneScan	Newbler Contigs	Cluster Contigs	Seeded Cluster Contigs	Equ-Cab2 Genes
28,789	70.1		√	√	√	58	412	7,760	20,559
817	2.0		√	√	×	37	360	219	201
652	1.6		√	×	√	11	641	0	0
5,391	13.1	e-value*	√	×	×	65	5,326	0	0
2,462	6.0	e-value∧	√	×	×	61	2,401	0	0
1,651	4.0		√	×	×	50	1,601	0	0
677	1.6	e-value*	×	×	×	13	299	79	286
287	0.7	e-value∧	×	×	×	9	273	0	5
314	0.8		×	×	×	4	308	0	2
41,040						308	11,621	8,058	21,053

* = e-value<1e^−20^,

∧ = e-value<1e^−4^.

Gene ontology analysis was performed on the library. Consistent with the utilization of CNS and immune tissues, a heavy bias towards those genes involved with the immune system, the CNS, and programmed cell death (apoptosis) was identified by GO analysis of the assembled contigs and genes. This methodology also allowed the identification of novel genes involved in the immune response and neurologically specific genes in equine tissues (Figure S1a, S1b).

The sequences were analyzed using the NCBI database and matching to known species in the database made. With an e-value≤10^−4^ for all databases, there were 39,257/41,040 accurate hits for both the NR and NT NCBI databases. For the NR database alone, there were 30,011 accurate hits with 26,955 ‘perfect’ (e-value = 0) hits, while for the NT database, there were 39,217 accurate hits with 32,825 ‘perfect’ hits. For both databases, matches to horses comprised the greatest number of hits with the species groups humans, primates, canines, and bovines containing the next greatest number of hits (Figure S2a, S2b).

All sequences that were not previously identified (novel) were analyzed. Of the 1,280 (3.1%) sequences not recognized by any of the equine databases, 709 (55.4%) were classified into gene ontology databases. With overlap, 579 could be classified into biological processes, 592 into cellular component, and 619 into molecular function. The average length of all these sequences was 595 base pairs with a range of 50–8,802 base pairs (Figure S3).

Finally, the sequences were compared to the human expressed sequence tag database (e-value≤10^−4^ ) (Table 4). A high degree of sequence homology between the human and equine transcriptome was found, with percentage matches of 69.27% (contigs), 78.13% (seed contigs), and 80.17% (singlets). Equine sequences demonstrated good match to the human EST database with an average percent identity of 90.17%, an average bit score of 512.91, and an average alignment length of 424.85.

**Table 4:** Summary of BLAST Analysis of Sequenced Equine Transcriptome to the Human Expressed Sequence Tag Database.

	Contigs	Seed Contigs	Singlets
Number of Matches E≤10^−4^	8050/11621	6296/8058	17127/21361
Percent Homology Match	69.27%	78.13%	80.17%
Average E value	1.44636E-05	2.38671E-06	8.09623E-06
Average Bit Score	189.0196894	907.9956004	519.9152099
% Identity	89%	91%	90%
Alignment Length	187.9218634	698.33831	435.6858761

Contigs, seed contigs, and singlets from this project were BLASTed against the human EST database. In total, 31,473 sequences matched to the human EST database (row 1) with an e-value≤10^−4^.

### Gene Expression Analysis

With respect to the experimental groups, three analyses were performed to determine if there was a difference in gene expression based on exposure status (the thalamic nuclei of the infected vs noninfected, nonvaccinated horses), immune status/survivorship (the thalamic nuclei of the infected vs infected, vaccinated horses), and location (the thalamic nuclei of the infected nonvaccinated cerebrum and infected nonvaccinated thalamus). These experimental groups and the tissue types tested are outlined in Table 1.

Loess normalization was performed on all arrays and confirmed by distribution analysis (Figure S4). For all arrays, the majority of variance was accounted for in the first three principal components (x, y, and z) with Eigenvalues (percents of variability) of the each component, 11.09 (30.81%), 4.94 (13.71%, and 3.21 (8.94%), respectively. In addition, the mean of the R^2^ was 0.939392 (range 0.8781–0.9871) for all arrays. A heat map and dendrogram was generated between all arrays (Figure S5).

Statistical analysis was performed using analysis of mean relative difference in gene expression. An ANOVA with interactions between treatment and location revealed significant differences in gene expression (p<0.05) for all comparisons, including those between exposure status, survival status, and CNS location. For the normal, non-challenged horses, there were 6,911 genes that showed showed significantly different levels of expression between cerebrum and thalamus after duplicate removal. Therefore, for the exposure and nonsurvival groups, only thalamus was compared. The three analyses were compiled and 3,421 of the same genes were significantly altered in all comparisons.

For exposure status, significant differences in gene expression in the thalamus were seen between nonvaccinated/exposed and normal, nonexposed horses for 9,020 genes (Table S5). When analyzed solely by fold change, 2,936 genes decreased by <−1.0 (395<−2.0) and 2,084 increased by >+1.0 (749>+2.0). For immune/survival status, significant differences in gene expression in the thalamus were seen between nonvaccinated/exposed horses (nonsurvivors) and vaccinated/exposed horses (survivors) in 7,395 genes (Table S5). In the nonvaccinated, nonsurvivors, 2,123 genes were decreased by <−1.0 (225<−2.0) while 1,800 were increased by >+1.0 (666>+2.0) compared to the vaccinated, survivors. Finally, where analyzed by location in the brain, significant differences in gene expression were seen between the cerebrum and thalamus of nonvaccinated horses exposed to WNV (location) for 7,649 individual genes (Table S5). For the location analysis, 2,053 genes were decreased by <−1.0 (609<−2.0) while 1,827 were increased by >+1.0 (406>+2.0).

#### Exposure Status

Gene ontology was explored for the exposure status. The first subhypothesis asked whether there was a difference in gene expression due to exposure to WNV between nonvaccinated horses exposed to WNV and normal horses not exposed to WNV. Genes that were found to have significant differences in expression were classified into the gene ontology (GO) categories of biological process, cellular component, and molecular function (Figure S6a). Within these categories, 2,022 genes mapped to components of transcription and RNA processing, 1,081 mapped to neurological categories, 983 genes mapped to immunological categories, and 420 mapped to cell death or apoptosis (430) (Figure S6b).

**Figure 1:**
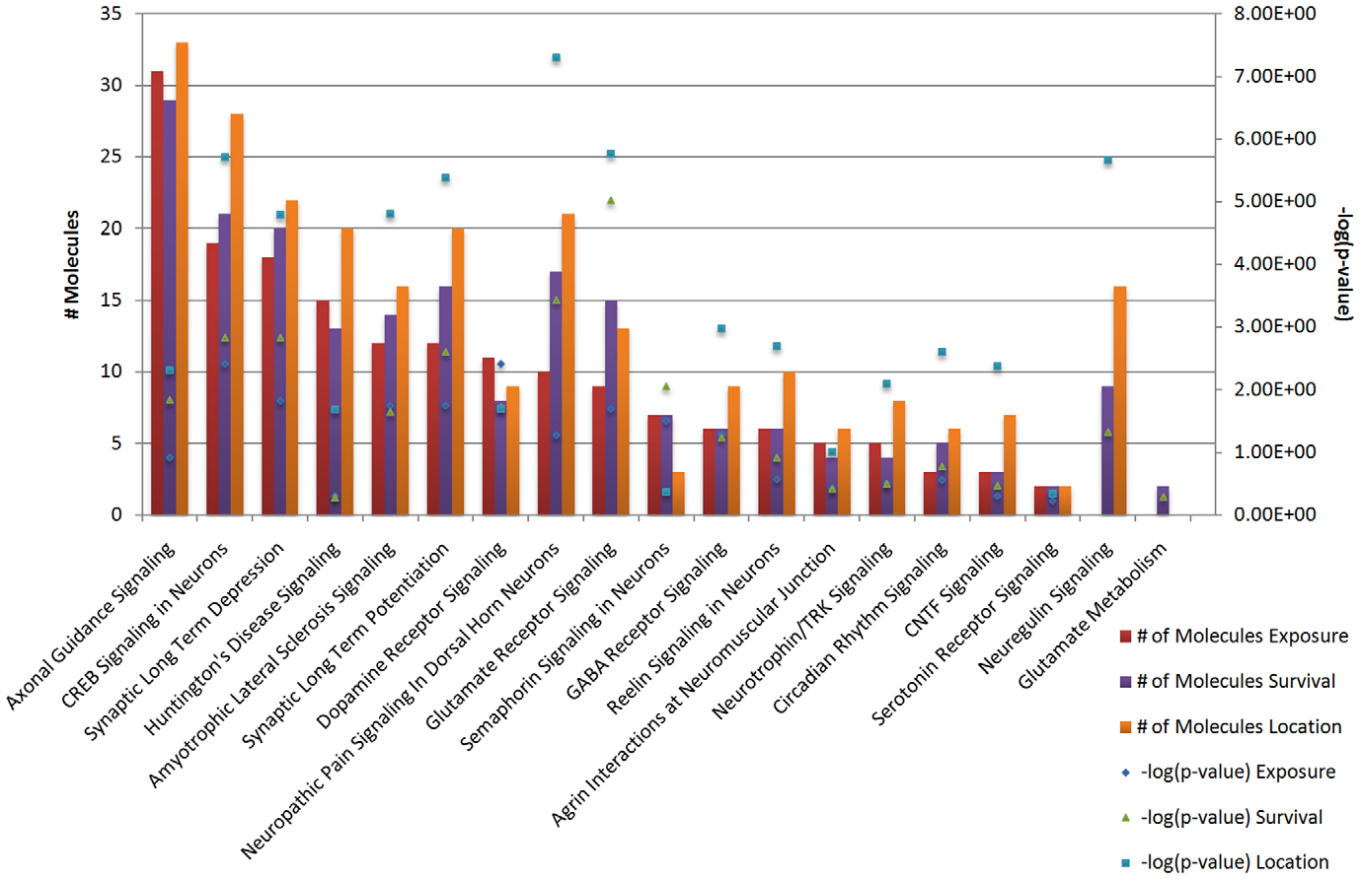
Neurological canonical pathways for all analyses. Canonical pathways identified as significant for each analysis were selected. There was a high degree of overlap between all three analyses in neurological pathways. The location analysis contained transcripts that mapped to the most neurological pathways. The green line represents significance. For the purposes of this study, ‘exposure’ represents the difference in gene expression between the nonvaccinated/exposed-normal, ‘survival’ represents the difference in gene expression between the nonvaccinated/exposed-vaccinated/exposed, and ‘location’ represents the difference in gene expression between the thalamus and cerebrum of the nonvaccinated/exposed.

**Figure 2:**
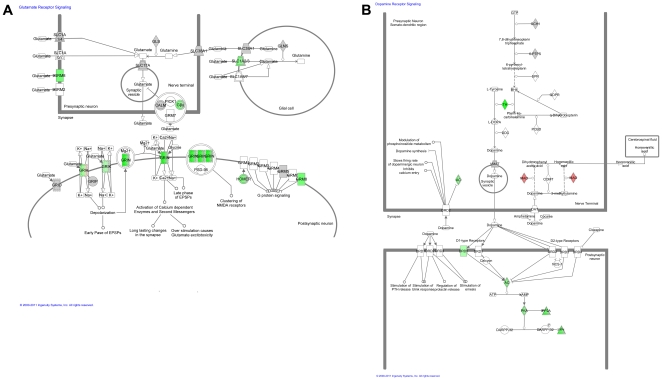
Glutamate and Dopamine Receptor Signaling Pathways. (A) The diagrams represents signaling in the synaptic cleft (square = pre-synaptic neuron, oval = post-synaptic neuron, circle = glial cell). Green represents downregulation of pertinent receptors, red represents upregulation of pertinent receptors. WNV induces downregulation of glutamate receptors on the post-synaptic cleft as well as glutamate uptake receptors on glial cells. (B) Dopamine receptor signaling pathway. The graphic shown is for the exposure analysis, but was similar in all analyses. Green represents down-regulation of transcripts, red represents upregulation. Dopamine receptors (DRD5) and downstream signaling pathways as well as enzymes that create dopamine (TH) were downregulated, while MAO (enzyme that degrades dopamine) was upregulated. This network was generated through the use of IPA (Ingenuity Systems, www.ingenuity.com).

Canonical pathways were first examined for interactions between multiple significant transcripts in the exposure status. The majority of pathways were involved with some aspect of cell signaling for all groups (Table S6). Seventeen neurological pathways were identified with significantly changed transcriptional levels when nonvaccinated horses exposed to WNV were compared with normal horses not exposed to WNV (Figure 1). The canonical pathways involved consisted mainly of neurotransmitter and signaling pathways with extensive involvement of glutamate receptor signaling (Figure 2a, Table 5) and dopamine receptor signaling (Figure 2b, Table 5). Neurological pathways containing the most molecules when the WNV infected horses were compared to the noninfected horses (15 or more) included axonal guidance signaling, CREB signaling in neurons, synaptic long term depression, and Huntington's disease signaling (Figure 1).

**Table 5:** Levels of Expression of Transcripts in Neurological Pathways of Note.

Entrez Gene Name	GenBank	Exposure	Exposure (p-value)	Survival	Survival p-value	Location	Location p-value
Adenylate cyclase 1 (brain)*	NM_174229			−1.789	1.78E-02	−1.898	1.25E-02
Adenylate cyclase 2 (brain)*	XM_851103	−1.683	−8.70E-08	−1.223	4.90E-06	−2.027	6.80E-09
Adenylate cyclase 5*	NM_183357	−1.258	2.07E-02	−1.379	1.19E-02		
Adenylate cyclase 8 (brain)*	XM_539166	−1.021	2.11E-02			−1.954	4.50E-05
Adenylate cyclase 9*	BC151229					−1.46	5.00E-04
Calcium/calmodulin-dependent protein kinase IV∧	XM_517873			−1.976	6.20E-03		
Dopamine receptor D5*	XM_604584	−1.097	1.72E-02				
GTP cyclohydrolase 1*	XM_846790			1.296	3.00E-03		
Glutaminase∧	AC005540			−1.009	9.00E-04		
Guanine nucleotide binding protein (G protein), beta polypeptide 1∧	BC004186	−1.638	7.80E-03				
Guanine nucleotide binding protein (G protein), gamma 5∧	BC003563					1.359	4.40E-05
Glutamate receptor, ionotropic, AMPA 1∧	XM_001169416	−1.398	1.34E-02	−1.057	5.00E-04	−1.498	3.00E-04
Glutamate receptor, ionotropic, AMPA 2∧	NM_000826			−1.505	4.00E-03	−1.643	3.90E-05
Glutamate receptor, ionotrophic, AMPA 3∧	NM_007325			−2.33	2.50E-03	−2.719	3.00E-04
Glutamate receptor, ionotrophic, AMPA 4∧	NM_000829	1.253	1.50E-02			−1.039	3.10E-03
Glutamate receptor, ionotropic, delta 2∧	AC022317			−1.581	1.00E-04		
Glutamate receptor, ionotropic, kainate 1∧	NM_000830	−1.098	2.59E-02	−1.933	1.57E-02	−2.626	3.00E-04
Glutamate receptor, ionotropic, kainate 2∧	XM_866973			−1.533	1.57E-02		
Glutamate receptor, ionotropic, N-methyl D-aspartate 1∧	AF015731			−1.949	6.00E-03	−1.487	1.99E-02
Glutamate receptor, ionotropic, N-methyl D-aspartate 2A∧	XM_547132	−2.836	1.00E-04	−1.631	2.85E-02	−2.369	1.00E-03
Glutamate receptor, ionotropic, N-methyl D-aspartate 2B∧	AC007535			−1.563	7.40E-03	−1.989	2.05E-02
Glutamate receptor, ionotropic, N-methyl-D-aspartate 3A∧	XM_862276					−1.032	1.10E-03
Glutamate receptor interacting protein 1∧	XM_001162097			−1.23	2.83E-02		
Glutamate receptor, metabotropic 8∧	AC079957	−1.856	1.14E-02				
Homer homolog 1 (Drosophila)∧	XM_001139767					−1.084	2.81E-02
Homer homolog 3 (Drosophila)∧	XM_541929	−1.718	1.10E-03	−1.134	2.31E-02		
Interleukin 4 induced 1*	AY358933	3.176	3.00E-05	3.265	2.00E-05	1.405	3.79E-02
Protein phosphatase 1, regulatory (inhibitor) subunit 14A*	XM_867134	−2.083	1.10E-03				
Protein phosphatase 1, regulatory (inhibitor) subunit 3C*	BT030698	−2.27	9.00E-04	−1.746	7.00E-12	−1.686	2.00E-11
Protein phosphatase 2 (formerly 2A), regulatory subunit B, beta isoform*	XM_001159292	−2.348	1.00E-04			1.14	3.49E-02
Protein phosphatase 2 (formerly 2A), regulatory subunit B, gamma isoform*	XM_001250700	−1.243	2.80E-03	−1.378	1.10E-03	−1.446	7.00E-04
Protein kinase, camp-dependent, catalytic, beta*	XM_862471			−2.225	7.00E-04		
Protein kinase, camp-dependent, regulatory, type II, beta*	XM_001148361	−1.627	2.20E-03				
Solute carrier family 1 (glial high affinity glutamate transporter), member 2∧	NM_004171	−1.639	1.53E-02	−2.869	7.80E-06	−2.278	2.00E-04
Solute carrier family 17 (sodium-dependent inorganic phosphate cotransporter), member 7∧	NM_001098046	−1.598	8.23E-02	−2.652	4.20E-03	−8.147	9.50E-08
Tyrosine hydroxylase*	BC149072	−2.857	1.00E-03				

* -Involved with Dopamine Signaling Pathway,

∧ - Involved with Glutamate Signaling Pathway.

**Figure 3:**
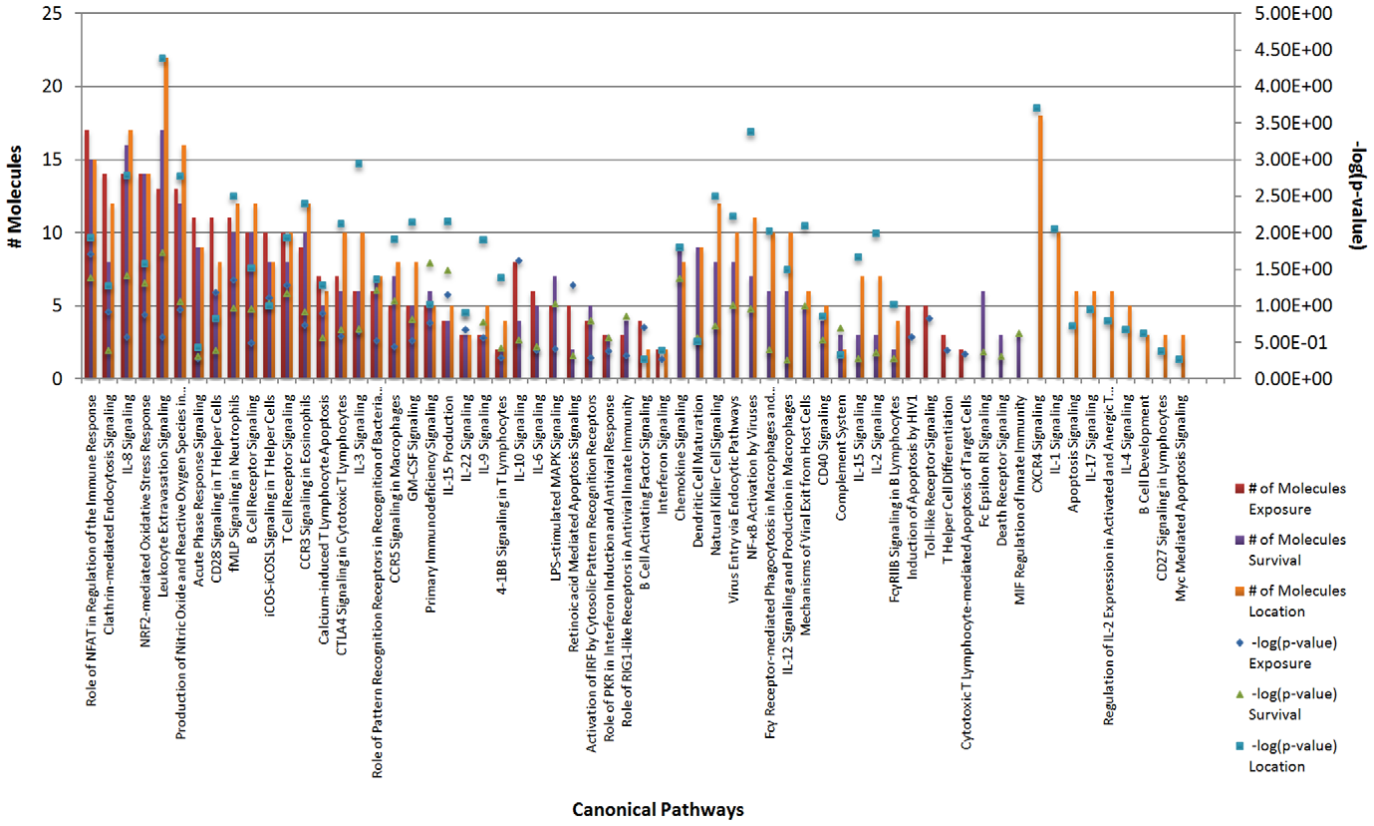
Immunological Canonical Pathways for All Analyses. The location analysis contained transcripts that mapped to the most immunological pathways. The green line indicates significance. For the purposes of this study, ‘exposure’ represents the difference in gene expression between the nonvaccinated/exposed-normal, ‘survival’ represents the difference in gene expression between the nonvaccinated/exposed-vaccinated/exposed, and ‘location’ represents the difference in gene expression between the thalamus and cerebrum of the nonvaccinated/exposed.

**Figure 4:**
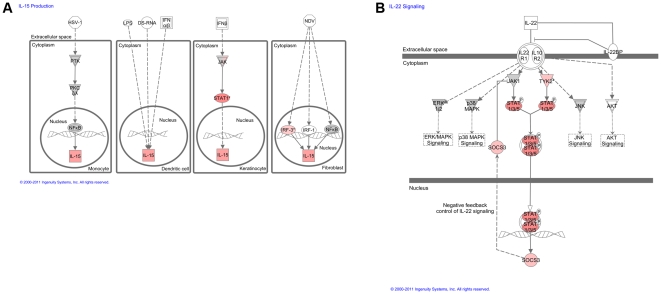
IL-15 Production Pathway and IL-22 Signaling Pathway. (A) The diagrams represent the different methods of IL-15 production. Green represents downregulation of pertinent molecules, red represents upregulation of pertinent molecules. WNV induces upregulation of the production of IL-15. (B) Red represents upregulation of gene expression, green represents down-regulation of gene expression. The JAK/STAT portion of the pathway is upregulated during viral infection, demonstrating an innate immune response. However, the SOCS3 molecule is also upregulated, indicating that infection with the virus may lead to subsequent suppression of the JAK/STAT pathway and evasion of the innate immune response. This network was generated through the use of IPA (Ingenuity Systems, www.ingenuity.com).

Regarding immunological canonical pathways and WNV exposure status, 47 pathways were identified (Figure 3) and involved in both the innate and adaptive response. Pathways in which there was upregulation in horses exposed to WNV included the IL-15 signaling pathway, the IL-22 signaling pathway, the IL-9 signaling pathway, and the Interferon Signaling Pathway (Figure 4a,b, Table 6). Multiple pathways involved in apoptosis were dysregulated (both up and down) in horses with exposure to WNV and these included the retinoic acid mediated apoptosis signaling, calcium-induced T lymphocyte induced apoptosis, cytotoxic T lymphocyte mediated apoptosis of target cells, induction of apoptosis by HIV1, and April mediated signaling.

**Table 6:** Levels of Expression of Transcripts in Immunological Pathways of Note.

Entrez Gene Name	GenBank	Exposure	Exposure p-value	Survival	Survival p-value	Location	Location p-value
Interleukin 15*	AK290619	2.369	7.40E-06	2.29	1.20E-05	2.004	4.00E-03
Interferon regulatory factor 3*	AK292027	1.31	1.80E-03	1.935	2.00E-05	1.086	3.32E-04
Janus kinase 1* ∧	XM_001161295					1.079	1.10E-03
Mitogen-activated protein kinase kinase 1* ∧	XM_612526					−1.4	3.79E-02
Mitogen-activated protein kinase 1* ∧	NM_002745			−1.095	6.40E-03	−1.061	9.50E-03
Protein inhibitor of activated STAT, 2∧	XM_612798			−1.014	1.20E-02		
Phosphoinositide-3-kinase, regulatory subunit 1 (alpha)* ∧	NM_181504	−1.062	3.00E-04			−1.339	1.00E-04
Phosphoinositide-3-kinase, regulatory subunit 2 (beta)* ∧	XM_847313			−2.44	2.40E-03	−2.341	1.15E-02
Phosphoinositide-3-kinase, regulatory subunit 3 (gamma)* ∧	XM_856294					1.537	2.43E-02
PTK2B protein tyrosine kinase 2 beta*	XM_543228			−1.029	8.60E-03	−1.545	1.10E-03
Suppressor of cytokine signaling 3∧	NM_174466	1.535	1.45E-02	1.809	4.60E-03		
Signal transducer and activator of transcription 1, 91kda* ∧	BC151378	3.021	3.00E-18	3.763	5.00E-20	2.384	1.00E-14
Tyrosine kinase 2* ∧	XM_590006	1.504	2.00E-04				

* - Involved with IL-15 Signaling Pathway,

∧ - Involved with IL-9;IL-22;JAK/STAT Signaling Pathways.

Genomic functions analysis links the top transcripts in each pathway to reported disease states and normal function. The functions were distributed amongst many analyses and, in particular, neurological, immunological, and cell death pathways (Table S7) were represented. In horses exposed to WNV compared to normal horses, four categories were identified involving neurological functions (2,326 transcripts), 10 categories were identified involving immunological functions (1,830 transcripts for exposure), and 1 category was identified as involving cell death (1,153 transcripts exposure).

**Figure 5:**
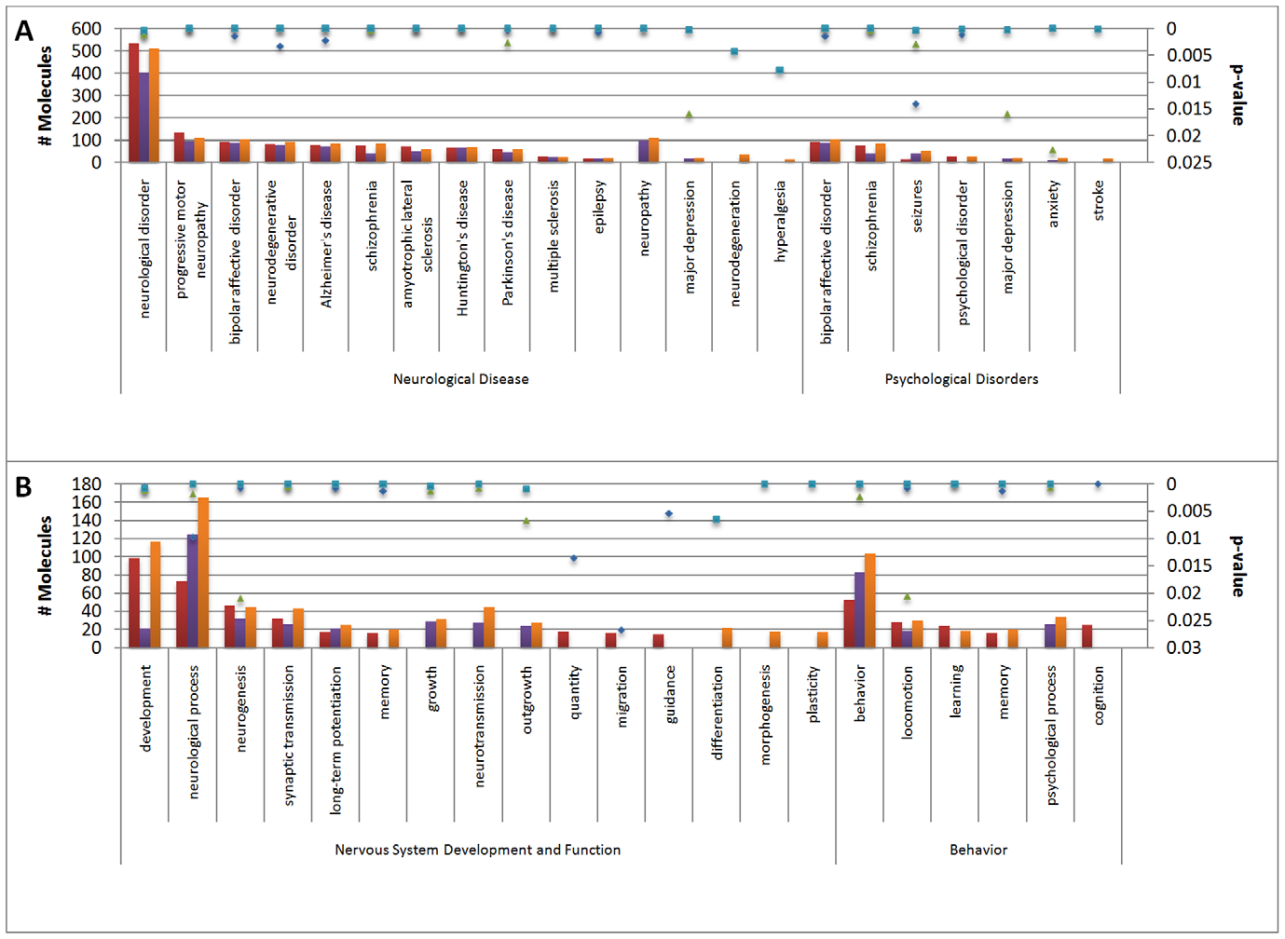
Neurological Functions for All Analyses. (A) Neurological disease and psychological disorders categories, and (B) Nervous system development and function and behavior categories. The majority of transcripts mapped to neurological disease. The red bars represent the exposure analysis, the purple bars the survival analysis, and the orange bars the location analysis. The diamonds represent the p-value for exposure, the triangles the p-value for nonsurvival, and the squares the p-value for location. For the purposes of this study, ‘exposure’ represents the difference in gene expression between the nonvaccinated/exposed-normal, ‘survival’ represents the difference in gene expression between the nonvaccinated/exposed-vaccinated/exposed, and ‘location’ represents the difference in gene expression between the thalamus and cerebrum of the nonvaccinated/exposed.

The genes in the functions from neurological categories were grouped mainly under neurological disease when compared to nervous system development and function, behavior, and psychological disease. When further analyzed by specific disease, genes mapped to mental disorders (including bipolar affective disorder, Alzheimer's, and schizophrenia), as well as degenerative neuropathies (including progressive motor neuropathy, Huntington's disease, Parkinson's disease, amytrophic lateral sclerosis, and multiple sclerosis) (Figure 5 a,b).

The functions involving immunological pathways were grouped with cell death/apoptosis for analysis. The most genes were categorized under inflammation (992 transcripts). Both innate (inflammatory response, antigen presentation, immune cell trafficking) and adaptive (humoral immune response, cell-mediated immune response, cytotoxicity, immune cell trafficking) aspects of immunity were identified. Cell death and apoptosis categories were also seen for the exposure analysis, with 1,299 total genes involved with cell death, and 1,006 total genes involved with apoptosis (Figure S7).

Transcripts that were increased in expression by 1-fold or more or decreased in expression by -1-fold or less and mapped to the IPA database were included. There were 176 neurological transcripts downregulated and 43 neurological transcripts upregulated (Table 7) for the exposure analysis. Transcripts involved with neurotransmitter pathways including glutamate receptor signaling (Figure 2a, Table 5) and dopamine receptor signaling (Figure 2b, Table 5) were of particular note. Catenin (cadherin-associated protein), delta 2 (neural plakophilin-related arm-repeat protein) (CTNND2) was also highly upregulated.

**Table 7:** Transcripts for all analyses mapped to neurological CPs.

	Exposure Decreased	Exposure Increased	Survival Decreased	Survival Increased	CNS Location Decreased	CNS Location Increased
Agrin Interactions at Neuromuscular Junction	1	4	2	2	2	4
Amyloid Processing	4	1	3	1	3	3
Amyotrophic Lateral Sclerosis Signaling	11	1	14	0	13	2
Axonal Guidance Signaling	25	6	22	7	19	10
CDK5 Signaling	11	0	9	0	10	2
Cholecystokinin/Gastrin-mediated Signaling	5	2	7	2	6	1
Circadian Rhythm Signaling	2	1	4	1	5	1
CNTF Signaling	1	2	2	1	4	3
CREB Signaling in Neurons	15	4	19	2	20	4
Docosahexaenoic Acid (DHA) Signaling			2	0	2	1
Dopamine Receptor Signaling	10	1	6	2	6	2
GABA Receptor Signaling	6	0	6	0	8	0
Glutamate Receptor Signaling	8	1	15	0	12	1
GNRH Signaling	12	3	11	1	13	2
Huntington's Disease Signaling	10	5	8	5	12	7
Melatonin Signaling	6	1	10	1	8	1
Neuregulin Signaling			6	3	10	6
Neuropathic Pain Signaling In Dorsal Horn Neurons	8	2	16	1	17	2
Neurotrophin/TRK Signaling	5	0	4	0	6	2
Reelin Signaling in Neurons	4	2	3	3	5	5
Regulation of Actin-based Motility by Rho			3	2		
Semaphorin Signaling in Neurons	6	1	5	2	2	1
Serotonin Receptor Signaling	1	1	0	2	1	1
Synaptic Long Term Depression	14	4	17	3	17	3
Synaptic Long Term Potentiation	11	1	15	1	17	1
Total	176	43	209	42	218	65

This table shows the number of transcripts that mapped to each pathway for all analyses. The majority of the transcripts demonstrated a decrease in expression values. Transcripts were included if they demonstrated a fold change >1 or <−1.

There were 176 immune transcripts downregulated and 130 immune transcripts upregulated (Table S8). The most notable was pentraxin 3 (PTX3), upregulated over 9-fold. Transcripts that mapped to specific immunological pathways of interest that were significantly changed in expression included those that mapped to the IL-15 pathway and those that mapped to the IL-9, IL-22, and JAK/STAT pathways (Figure 4a,b, Table 6). Transcripts involved in apoptosis were also upregulated in WNV infected horses compared to normal horses, including poly (ADP-ribose) polymerase family member 14 (PARP), caspase 4 (CASP4) retinoid receptor (RXR), and retinoic acid nuclear receptor (RAR).

#### Immune/Survivor Status

Gene ontology was investigated for the immune/survivor status. Horses that survived infection (all vaccinated before experimental infection) were compared to horses that succumbed to grave neurological diseases (no vaccine before experimental infection). Because of experimental design to allow for assessment of survivorship, nonsurviving horses were analyzed at an average of 7–9 days post-infection compared to survival horses at 21 days post-infection. Similar to previous analysis performed between exposed and nonexposed horses, genes that were found to be significantly different in expression belonged to three major gene ontology (GO) categories (Figure S6a). The most genes mapped to GO processes of transcription/RNA processing (1,864) with the second most genes mapping to immunological categories (850). Genes also mapped to neurological categories (840) and cell death/apoptosis (338) (Figure S6b).

For this analysis, the majority of canonical pathways engaged cell signaling for a variety of cell types, functions and transcripts (Table S6). Ten of the top 25 pathways (based on the p-value) were classified as neurological pathways composed of 156 transcripts. None of the top 25 pathways were identified as immunological pathways.

The neurological canonical pathways were analyzed for nonsurvivorship and 19 pathways were identified (Figure 1). Like the analysis for exposure status, specific neurological pathways that demonstrated dysregulation included neurotransmitter pathways and signaling pathways with glutamate receptor signaling (Figure 2a, Table 5) and dopamine receptor signaling (Figure 2b, Table 5) highly represented once again. The pathways with the most molecules ( greater than 15) involved included axonal guidance signaling, CREB signaling in neurons, synaptic long term depression, neuropathic pain signaling in dorsal horn neurons, synaptic long term potentiation, and glutamate receptor signaling.

Forty-nine pathways involved in both the innate and adaptive immunity were identified as associated with immune/survivorship status (Figure 3). Some of the major immune pathways that were upregulated included the IL-15, IL-22, IL-9, and IFN signaling pathways (Figure 4a,b, Table 6). Multiple pathways involved in apoptosis were also dysregulated and these included the retinoic acid mediated apoptosis signaling, calcium-induced T lymphocyte induced apoptosis, and death receptor signaling.

Five neurological function categories involving 2,246 transcripts were identified as significantly altered when surviving horses were compared with nonsurviving horses, while nine categories were identified involving immunological functions (1,542 transcripts), and one category was identified involving cell death (1,082 transcripts exposure) (Table S7).

In the analysis of specific neurological categories, more genes grouped under neurological disease when compared to nervous system development and function, behavior, and psychological disease. When further analyzed by specific disease, genes mapped to the similar mental and degenerative disorders identified by the previous analysis (Figure 5 a,b).

Functions similar to those identified in the previous exposure analysis were noted when deeper analyses of immunological and apoptosis functions was performed. Genes were mostly commonly categorized under inflammation (832 transcripts), with both innate and adaptive immune functions identified. In total, 476 total genes were involved with cell death, and 340 genes were involved with apoptosis (Figure S7).

Individual significantly upregulated and downregulated transcripts were analyzed for their association with nonsurvival (naïve horses infected with WNV). A total of 209 neurological transcripts were decreased in expression and 42 neurological transcripts were increased in expression (Table 7). Similar to previous analyses, these transcripts were primarily involved with neurotransmitter pathways including glutamate and dopamine receptor signaling (Figure 2ab, Table 5).

Further analysis of the transcripts involved in the immune response in the nonsurvivors identified 215 downregulated and 116 upregulated immune transcripts (Table S8). The transcript increased most in expression was PTX3 exhibiting a 7.7-fold increase in nonsurviving horses compared to surviving horses. Transcripts that mapped to separate immunological pathways that were significantly changed in expression included those that mapped to the IL-15, IL-9, IL-22, and JAK/STAT pathways (Figure 4a,b, Table 6). Transcripts predominantly involved in apoptosis were also upregulated for the nonsurvivors and examples included (PARP) and caspase 4.

#### CNS Location

Gene ontology was analyzed for CNS location. The third subhypothesis asked whether there was a difference in gene expression due to location in the brain during WNV infection between thalamus and cerebrum, and for this only infected, nonvaccinated horses were analyzed. The transcripts were mapped to the three major GO categories (Figure S6a). Most genes mapped to GO processes of transcription/RNA processing (1,664) with the second most genes mapping to immunological categories (798). Genes also mapped to neurological categories (447) and cell death/apoptosis (349) (Figure S6b).

Canonical pathways analysis was performed on the data in the location status. Similar to previous analyses, the majority of pathways affected and differentially expressed were involved with cell signaling (Table S6). Seven of the top 25 pathways were identified as important in neurological processes (125 transcripts) while two of the top 25 pathways were identified as immunological processes (40 transcripts).

Sixteen canonical pathways were significantly affected based on differences in CNS location (Figure 1). Like the exposure and survivor analysis, neurotransmitter pathways and signaling pathways were heavily involved, including glutamate receptor signaling (Figure 2a, Table 5) and dopamine receptor signaling (Figure 2b, Table 5). Pathways with greater than 15 molecules involved included axonal guidance signaling, CREB signaling in neurons, synaptic long term depression, neuropathic pain signaling in dorsal horn neurons, Huntington's disease signaling, synaptic long term potentiation, and neureglin signaling (Figure 1).

Forty-eight immunological canonical pathways (Figure 3) involving both the innate and adaptive response were detected as significantly involved. The immune pathways that were upregulated (Figure 4a,b, Table 6) included the same previously identified signaling pathways (IL-15, IL-22, IL-9 and IFN). Multiple pathways involved in apoptosis were also dysregulated in the location analysis. These included the retinoic acid mediated apoptosis signaling, calcium-induced T lymphocyte induced apoptosis, cytotoxic t lymphocyte mediated apoptosis of target cells, induction of apoptosis by HIV1, and apoptosis signaling, and myc mediated apoptosis signaling.

Five categories were identified involving neurological functions (3,242 transcripts), ten categories were identified involving immunological functions (1,558 transcripts), and one category was identified as involving cell death (719 transcripts) (Table S7). The further analyses of specific neurological function were similar to that mapped for both the exposure and immune status analyses (Figure 5 a,b).

The functions involving immunological pathways were grouped with cell death/apoptosis for analysis. Like the previous analyses, most genes were categorized under inflammation (834 transcripts), with involvement of both innate and adaptive immunity. Cell death and apoptosis categories were also seen for the location analysis, with 210 total genes involved with cell death, and 184 total genes involved with apoptosis (Figure S7).

Neurological transcripts were significantly changed in expression (Table 7). A total of 176 transcripts were downregulated and 43 transcripts were upregulated. Transcripts of note were similar to the previous analyses. This included a decrease in transcripts involved with glutamate signaling and dopamine signaling pathways (Figure 2a,b, Table 5). The protein CTNND2 was also highly upregulated.

In total, 266 immune transcripts were downregulated, while 210 transcripts were upregulated (Table S8). PTX3 was upregulated over 4-fold. Other immunological transcripts significantly changed in expression were similar to the analyses involving exposure and immune status. These included those that mapped to the IL-15 pathway and those that mapped to the IL9, IL22, and JAK/STAT pathways (Figure 4a,b, Table 6). Apoptotic transcripts were also upregulated in the location analysis and were similar to that of immune/survivorship status, including poly (ADP-ribose) polymerase family member 14 (PARP), and caspase 4 (CASP4).

#### Comparison of Histopathological Data and Clinical Signs with Gene Expression Data

All horses were evaluated for clinical signs and neurological exams were performed. Infected, non-vaccinated horses (exposure group) all demonstrated ataxia and muscle fasciculations. The majority of these horses also demonstrated a change in mentation (4/6) and/or paresis (5/6) (Table S9). All of the exposed naïve horses developed clinical signs, half developed a fever, and all were humanely euthanized (Table 8). Infected vaccinated horses and normal control horses did not develop clinical signs, signs of neurological disease, or fever (Table 8). Viremia was detected in non-vaccinated exposed horses from days 1–5 post infection, but not in the vaccinated exposed horses [22].

**Table 8:** Summary of outcomes of each treatment group analyzed by CNS microarray.

Vaccine	Clinical Signs	Fever^b^	Survival^c^	Virus Isolation	Histopathology
**Infected Vaccinated (6)**	0/6	0/6	0/6	0/6	1/6^e^
**Infected Nonvaccinated (6)**	6/6^a^	3/6	6/6	6/6	6/6^d^
**Noninfected (6)**	0/0	0/0	0/0	0/0	0/0

a Moderate or severe signs in at least one of the following categories for at least two days: mentation, paresis, fasciculation, or ataxia.

b Body temperature ≥39.2°C (102.5°F).

c Death due to development of WNV disease severe enough to require euthanasia for humane reasons.

d Encephalitic horses in the control group had moderate or severe encephalitis on histopathology.

e Mild inflammatory histopathologic changes in neural tissues vaccinated horses.

For histopathological analysis, all horses in the non-vaccinated exposed group demonstrated moderate or severe encephalitis on histopathology (Table 8). This pathology was graded according to the degree of gliosis and/or perivascular cuffing and was more severe in the thalamus of these horses compared to the cerebrum (Table 9). Mild signs of encephalitis were seen in 1 out of the 6 infected, vaccinated horses (Table 8). These inflammatory changes were more severe in the thalamus than in the cerebrum (Table 9). No abnormal histopathology was seen in the cerebrum or thalamus of the normal control horses. The severity of the clinical signs and histopathology in the non-vaccinated horses exposed to WNV, compared to the exposed vaccinates and normal controls, is consistent with the changes in gene expression seen between the analyses of exposure and survival. This is likewise true for the location analysis when examining the histopathological differences between the thalamus and cerebrum of the exposed, non-vaccinated horses.

**Table 9:** Histopathology on individual animals.

Treatment	Animal	Pathology Score in Cerebrum	Pathology Score in Thalamus
**Infected Vaccinated**	**Horse 1**	0(18)	0(17)
**Infected Vaccinated**	**Horse 2**	1(13)	1(12)
**Infected Vaccinated**	**Horse 3**	0(10)	3(43)
**Infected Vaccinated**	**Horse 4**	0(2)	1(17)
**Infected Vaccinated**	**Horse 5**	0(7)	0(3)
**Infected Vaccinated**	**Horse 6**	0(2)	0(10)
**Infected Nonvaccinated**	**Horse 7**	2(40)	29(93)
**Infected Nonvaccinated**	**Horse 8**	4(58)	18(100)
**Infected Nonvaccinated**	**Horse 9**	6(60)	11(82)
**Infected Nonvaccinated**	**Horse 10**	7(75)	17(83)
**Infected Nonvaccinated**	**Horse 11**	0 (8)	10(32)
**Infected Nonvaccinated**	**Horse 12**	1(10)	7(50)
**Not Infected**	**Horse 13**	0	0
**Not Infected**	**Horse 14**	0	0
**Not Infected**	**Horse 15**	0	0
**Not Infected**	**Horse 16**	0	0
**Not Infected**	**Horse 17**	0	0
**Not Infected**	**Horse 18**	0	0

This table shows the lesions that were quantified in the pons, medulla, cervical cord and lumbar cord. Briefly cross sections of these areas were examined for the presence of gliosis and perivascular cuffing. One section each was evaluated for the pons and medulla. Two sections were evaluated for each area of the spinal cord. Total numbers of glial nodules were counted in each section. If more than one section was evaluated the counts for these sections were averaged. For pervascular cuffs, 3 areas were examined in each section and 10 vessels were counted in each area. The number of vessels that contained inflammatory cells was divided by the number 10. Each area per section was averaged.

### Array Validation

The correct sequences (checked against the sequences from the transcriptome) were identified for the primer pairs β-actin, 2′5′OAS, CC1R, IL-6, DEADBox60, Defensin β 4, and TNF-α. β -actin was included as the ‘house-keeping gene’, and the other genes were significantly up-regulated. Primer efficiencies were established for all primer pairs using standard curves analysis and efficiency calculation, with efficiencies ranging between 85 and 97%. Real time relative quantitative PCR was then performed on the thalamus from 6 of the vaccinated and exposed horses and 6 of the non-vaccinated and nonexposed horses, with β actin as the endogenous control (Table 10). There was a relative increase in expression for all primer pairs which correlated with the microarray data.

**Table 10:** Validation of the array.

	Nonvaccinate average relative expression QPCR	Vaccinate average relative expression QPCR	Average expression nonvaccinate: vaccinate QPCR	Nonvaccinate: vaccinate array expression
2′5′OAS	+1.689667	−0.71533	+2.4050	+6.539663
Complement Component 1 r	+2.0895	−0.2185	+2.3080	+1.886843
DEADBox60	+1.7625	−0.895	+2.6575	+5.651655
Defensin B4	+0.365333	−1.907	+2.2723	+6.99401
IL-6	+1.342833	−0.56933	+1.9122	+5.97945
TNF	+0.649	−0.90583	+1.5548	+3.471118

Comparison of the relative expression levels between the nonvaccinate and vaccinate thalamus and QPCR to array platforms.

Microarray probe sequences were analyzed by comparison to the most recent version of the EqCab2 genome using the basic local alignment search tool (BLAST, Fisher Cluster, University of Florida, Gainesville, FL) to determine the accuracy of each sequence to detect single genes as opposed to gene families. In total, 42,843 oligonucleotide probes were analyzed and 40,113 probes matched to one sequence with 100% identity (93.6%). Of these, 3,700 (9.2%) matched more than once to a genomic sequence implying possible binding to a gene family. The majority of these which matched to multiple sequences were identified as belonging to one chromosome. In addition, 2,687 probes matched at <100% identity (average 97.5% identity). Forty-three probes did not match, and were most likely present as controls since the sequences could not be detected in the sequenced library.

## Discussion

This project sequenced all levels of equine brain and spinal cord transcriptome to analyze global gene expression in the CNS of natural, outbred equine hosts during grave WNV encephalitis. The data generated from this project provides invaluable insight into WN encephalitis in horses and possibly humans, and is a useful platform for future studies for both pathological and non-pathological applications. In the sequencing and analysis of the transcriptome, 41,040 sequences were identified by BLAST analysis in 5 sequence databases. There was overall consensus amongst the NCBI databases as to the hits on species, with the vast majority of sequences matching to the horse. Further analysis of the sequenced transcriptome revealed that 9,504 of the identified sequences were missed by equine predicted databases, and 1,280 of the identified sequences have yet to be discovered in the equine genome project. This is most likely due to the incomplete annotation of the equine genome and differential expression of the equine brain in disease allowing for detection of rare transcripts. Since other species' genomes have undergone more comprehensive annotation and analysis, transcriptomic sequences that should be recognized by the equine databases may be recognized in other organisms. Another possibility for the discrepancy could be differences between the equine genome and transcriptome (i.e. splice variants). These issues are likely to improve with time and more tissue specific transcriptome analyses. This portion of the project also demonstrated high sequence homology between the equine and human EST database, showing that the horse may be useful in the study of the human organism.

The main hypothesis investigated was that there are families of genes that are changed in a consistent manner in horses undergoing WN encephalitis. In the analysis of the microarray data, three subhypotheses were investigated to explore whether there was a difference in gene expression based on the state of exposure, immunity/survival, and location in the CNS. Because there was high amount of overlap in our findings from these analysis, either these findings support a generalized model of WNV encephalitis based on exposure status, recovery, and CNS pathology, or the state of WN infection without regard to immunity and recovery has been primarily modeled. Alternatively, it is possible that immunity from WN encephalitis after exposure is similar to a completely naïve, nonexposed state. Additionally, it is likely that the time of sample collection (21 days for vaccinated/exposed horses, 7–9 days for nonvaccinated/exposed horses) influenced gene expression in immune horses. However, overall, it appears that horses that are exposed to WNV demonstrate similar changes in gene expression, which are highlighted by the changes in the thalamus. Finally since the results appear to be location dependent with the majority of differentially expressed genes in the gray matter coinciding with disease/survivorship state, study of WNV in the brain is topical and likely best elucidated in a tissue specific manner.

A total of 17 neurological canonical pathways were identified across the three analyses, the majority of which involved cell signaling within the nervous system. Neurotransmitter pathways were one of the top dysregulated pathways for all groups, including glutamate and dopamine pathways. Glutamate is the primary excitatory neurotransmitter in the neurological system. Previous work has demonstrated that an excess of glutamate at the synaptic cleft can lead to apoptosis of neurons through glutamate excitotoxicity as a cause of pathology in many neurological conditions [23], [24], [25], [26], [27]. In this study, the nonvaccinated group of horses exposed to WNV demonstrated gene expression changes consistent with glutamate excitotoxicity when compared to both the vaccinated and normal (non-exposed) control horses. This was also true when comparing the thalamus of the non-vaccinated horses exposed to WNV to the cerebrum of these same horses. Changes consistent with glutamate toxicity included a decrease in the expression levels of NMDA glutamate receptors, metabotropic glutamate receptors, kainate glutamate receptors, ionotropic glutamate receptors, and glutamate clearance receptors. Infection with WNV may lead to a downregulation of glutamate receptors on the post-synaptic neuron as well as glutamate uptake receptors on glial cells, leading to an increase in glutamate levels in the synaptic cleft and pathology associated with glutamate excitotoxicity.

Dopamine was another neurotransmitter pathway that was significantly changed in all three groups. Dopamine is a stimulatory neurotransmitter that functions, among other things, in the control of voluntary movement [28]. In the nonvaccinated group of horses exposed to WNV, a decrease was seen in the expression levels of dopamine receptor D5 as well as the downstream affector transcripts AC, PC, and PP. In addition, tyrosine hydroxylase, which catalyzes the conversion of tyrosine to dopamine, was downregulated. The expression of monoamine oxidase (MAO), which functions to breakdown dopamine, was increased in the nonvaccinated exposed group. Thus exposure to WNV may lead to a decrease in dopaminergic receptors and subsequent downstream signaling, a decrease in enzymes to create dopamine, as well as an increase in MAO. This results in a total decrease in available dopamine, which may explain many of the clinical signs seen in WNV infection that seem to mimic human disorders such as Parkinson's disease. Further study involving the detection and quantification of the transcripts from neuronal cells infected with WNV associated with the glutamic and dopaminergic pathways is necessary before any firm conclusions can be drawn.

Clinical neurological disease in horses caused by WNV is characterized by a combination of spinal cord, midbrain/hindbrain, and mentation abnormalities, with long-term residual neurological deficits [29]. The clinical signs seen in horses during WN infection mimic many of the clinical signs seen in some human neurological disorders, such as Parkinson's disease, progressive motor neuropathy, Huntington's disease, neurodegeneration, amyotrophic lateral sclerosis, and multiple sclerosis. For this study, it was found that many of the pathways and transcripts previously shown to be dysregulated during these diseases are also abnormally expressed during WNV infection in horses. Thus neurological infection with WNV in horses appears to mimic many of the seemingly non-infectious neurological disorders seen in man on both the clinical disease scale and the transcriptomic level. It is possible that seemingly non-infectious neurological disease may have an infectious origin, or that the brain can only behave and react in a certain manner no matter the stimulus or insult. This study demonstrated that infection with WNV leads to dysregulation in known neurological disease gene pathways, including those involved with neurotransmission and downstream signaling. This corresponds with clinical signs of disease in affected hosts, and also suggests a correlate between the neuropathology induced by viral infection of the CNS and the neuropathology seen in non-infectious neurological disease.

The similarities between the three analyses can also be seen when examining the immunological pathways and functions. This study demonstrated that changes in both the innate (inflammatory response, antigen presentation, immune cell trafficking) and adaptive (humoral immune response, cell-mediated immune response, cytotoxicity, immune cell trafficking) immune pathways are present during WNV infection. In general, the majority of immune transcripts and pathways were decreased in expression in the nonvaccinated horses exposed to WNV, providing evidence of downregulation of a balanced immune response during WNV infection at the peak of clinical disease.

In contrast, some immune pathways, such as the interleukin-15 signaling pathway, were upregulated during WNV infection in nonvaccinated horses exposed to WNV. IL-15 has been shown to be particularly important in providing a protective immune response to viral infection [30], [31], [32], [33]. For all three analyses, IL-15 was upregulated over 2-fold, as well as the transcription factor STAT1, which was upregulated over 2–3 fold. Interestingly, the downstream elements of IL-15 were downregulated in the unvaccinated horses exposed to WNV. The virus, either directly or indirectly, may be blocking the downstream effector elements of the IL-15 pathway to prevent the host immune response to the virus. There could also be other elements in the IL-15 pathway that are not yet elucidated. It is also possible that this finding is only a reflection of the timing when the naïve horses exposed to WNV were euthanized (at the onset of clinical signs) and a beneficial response from IL-15 to viral infection could not be realized in these horses. Thus it appears that IL-15 is upregulated in response to WNV infection, and while it may play a key role in recovery from viral infection, its dysregulation may be a key component of the immunopathology of this disease. Continued work targeting the quantification of IL-15 levels during viral infection at different time points is necessary for further clarification of this data.

Other pathways that were upregulated in non-vaccinated horses exposed to WNV were the IL-22, the IL-9, and the interferon signaling pathways with IL-22 and IL-9 activating similar transcripts. Both of these pathways activate JAK and TYR transcripts, which in turn phosphorylate and activate STAT (Signal Transducers and Activators of Transcription)- specifically STAT1, STAT3, and STAT5. These STAT transcripts induce the expression of ISGs (interferon stimulated genes) through a variety of mechanisms, and lead to the induction of an innate antiviral response [34]. As expected, expression of these JAK/STAT transcripts is upregulated during WNV infection in the unvaccinated horses exposed to WNV. Of interest as well is the finding that the SOCS3 (suppressor of cytokine signaling 3) is also upregulated in the exposure and survival analyses. SOCS3 functions as a negative feedback inhibitor on the JAK/STAT pathway, thereby inhibiting the innate immune response [35]. Transcripts of SOCS1 and SOCS3 have been shown to increase during WNV infection of the murine brain [36]. Upregulation of SOCS3 allows the virus to escape the innate immune response and has also been shown to lead to chronic infection and inflammation. This is further supported by decreased expression of the transcriptional regulators ASB1 and ASB5, which function to suppress SOCS expression. Thus it is possible that while the JAK/STAT pathway is upregulated in response to WNV infection for the activation of innate immunity, WNV may induce the expression of the SOCS3 molecule to suppress this pathway and evade the innate immune response.

The transcript increased the most in expression for all analyses was pentraxin 3 (PTX3). This was the case for all analyses when comparing the unvaccinated exposed horses to the vaccinated exposed and normal horses, as well as the thalamus to the cerebrum (thus the highest levels of expression were in the unvaccinated controls). This molecule has many functions, including an integral role in the pathway of pattern recognition receptors in recognition of viruses and bacteria [37], [38]. This gene is induced by IL-1b, and functions in the phagocytosis and opsonization of antigens, as well as in the inflammatory response. Thus infection with WNV and recovery from disease may be associated with an increase in this molecule that plays an integral role in innate immunity. Another transcript that was highly increased in expression for all analyses (greatest level of expression in the non-vaccinated controls compared to other groups and in the thalamus compared to the cerebrum) was the brain specific molecule CTNND2, which functions to connect cell junctions and cytoskeletal architecture with signaling pathways [39]. This may provide evidence that dysregulation of neurological tissue, such as that induced during WNV infection, leads to re-arrangement of neuronal architecture and the induction of signaling. This may also be important in viral entry into the cell. Apoptotic transcripts that were upregulated in all analyses included PARP and CASP4. Some apoptotic transcripts were upregulated in only the exposure analysis, including RXR and its receptor RAR. Understanding which transcripts are upregulated or downregulated during viral infection provides a glimpse into the affect of the virus on individual transcripts and, with further studies, could lead to the elucidation of many unanswered questions. These could include an understanding of how the virus invades the cell, as well as which cell molecules the viruses uses for replication, transcription, and translation.

The changes in gene expression data were also compared to clinical signs and histopathological data. Horses that were infected and not vaccinated demonstrated abnormal neurological and clinical signs as well as viremia, warranting humane euthanasia by day 9 of the study. On histopathological analysis, these horses also demonstrated moderate to severe signs of encephalitis, worse in the thalamus than in the cerebrum. This was in comparison to the vaccinated, exposed horses and normal horses that did not display any abnormal clinical or neurological signs and did not have a detectible viremia. Only a mild encephalitis was seen in 1 of the 6 vaccinated and exposed horses, with no abnormal histology in the normal controls. This correlates well to the gene expression data, as the non-vaccinated exposed horses demonstrated the greatest changes in gene expression when compared to the other groups of horses. The severity of the clinical signs and histopathology in the non-vaccinated horses exposed to WNV, compared to the exposed vaccinates and normal controls, is consistent with the changes in gene expression seen between the analyses of exposure and survival. This is likewise true for the location analysis when examining the histopathological differences between the thalamus and cerebrum of the exposed, non-vaccinated horses.

In summary, the microarray proved to be a useful tool to understand changes in gene expression patterns during WNV infection utilizing a custom fabricated microarray enriched for neurological and immunological sequences for study in the clinically affected host. Significant changes were identified in neurological, immunological, and apoptotic pathways with associations made between viral encephalitis and non-infectious neurological disease based on a systems biology approach. This information will eventually be integrated with other components of a systems biology approach, combining interdisciplinary scientific fields to validate these findings. This information could eventually be used to combat not only outbreaks of WNV, but also as a model to understand and reduce the impact of viral encephalitis in general.

## Supporting Information

Methods S1 **Supplemental methods information, including information on RNA extraction and quality assessment, the construction and normalization of the cDNA library, production of samples for analysis on the array, hybridization and scanning of the arrays, and microarray validation.**
(DOCX)

Figure S1 **Gene ontology classification of physiological processes.** These categories were included under biological process. The majority of genes were involved with neurophysiological processes (1,920) and the immune response (1,272).
(TIF)

Figure S2 **Sequence count by species group for the NCBI NR/NT databases.** A.) Sequence count for the NR database. The majority of sequences mapped to the horse, with other prominent groups including the human, primate, canine, and bovine. B.) Sequence count for the NT database. The majority of sequences in this database also mapped to the horse, with other prominent groups including the human, primate, canine, and bovine.
(TIF)

Figure S3 **Average length of novel sequences.** The majority of sequences annotated were less than 1000 base-pairs, with an average length of 595 base-pairs.
(TIF)

Figure S4 **Box plots for Loess normalization.** The green line indicates the mean of all arrays after normalization, while the red boxes indicate the range of response, the red lines in the boxes the median of each array, and the extended red lines standard deviations. All arrays normalized correctly.
(TIF)

Figure S5 **Heat Map and Dendrogram of All Arrays Demonstrating Similarity in Gene Expression.** Dark red indicates a high degree of similarity, while blue indicates a low degree of similarity.
(TIF)

Figure S6 **Number of Genes that Mapped to GO Categories for All Analyses and Distribution of Genes Among GO Categories.** A. The distribution of genes is relatively even, with slightly more genes overall in the exposure analysis compared to the nonsurvival and location analysis. B. Most genes for all analyses were classified under transcriptional categories, with neurological categories containing the second highest number of genes. For the purposes of this study, ‘exposure’ represents the difference in gene expression between the nonvaccinated/exposed-normal, ‘survival’ represents the difference in gene expression between the nonvaccinated/exposed-vaccinated/exposed, and ‘location’ represents the difference in gene expression between the thalamus and cerebrum of the nonvaccinated/exposed.
(TIF)

Figure S7 **Immunological and cell death/apoptosis functions for all analyses.** The majority of all transcripts mapped to the exposure analysis. Both innate and adaptive immune categories are present, as well as both cell death and apoptosis. For the purposes of this study, ‘exposure’ represents the difference in gene expression between the nonvaccinated/exposed-normal, ‘survival’ represents the difference in gene expression between the nonvaccinated/exposed-vaccinated/exposed, and ‘location’ represents the difference in gene expression between the thalamus and cerebrum of the nonvaccinated/exposed.
(TIF)

Table S1 **Probe groups for inclusion on the microarray.** Probes were included on the array once in the ‘plus’ (5′–3′) orientation (Annotated). Probes were individually selected to be included twice on the array (Important analyse) and included sequences involved in neurological, immunological, and transcriptional processes, as well as cell death. Probes that were determined to be correctly oriented in the ‘minus’ (3′–5′) direction were included in Annotated_minus. Unannotated probes and probes recovered from the EqCab2 genome sequencing project were also included. 250 Agilent controls were incorporated.
(DOCX)

Table S2 **Average Nucleic Acid Quality Data for All Samples.** Quality assessment was performed on all RNA samples used to create the cDNA library. The quality of the RNA was checked using spectrophotometric technology (ND-1000, Nanodrop Technologies, Wilmington, DE) and a microfluidics platform (Agilent 2100 Bio-analyzer, Santa Clara, CA). Only samples with a RIN>6 and a 260∶280 ratio >1.80 were used in the experiment. Quality assessment was also performed on the cDNA libraries including the 260∶280 ratio (>1.80) and concentration measurements.
(DOCX)

Table S3 **Data from 454 Sequencing Runs Newbler Assembler.** Newbler assembly software was first used to assemble the sequences. 514,462 sequences were assembled from a total of 49,857,586 bases after linker contamination was removed. Sequences that could be linked from beginning to end were classified as ‘fully assembled reads’, sequences that were shown to have some association were classified as ‘partially assembled reads’, standalone sequences coding once for areas of individual genes were classified as ‘singletons’, standalone sequences coding for areas of individual genes at a frequency of greater than 5% were classified as ‘repeats’, and standalone sequences coding for areas of individual genes at a frequency of less than 5% were classified as ‘outliers’. From this data, 16,895 contigs (sets of overlapping DNA sequences) composed of 4,720,747 bases were assembled.
(DOCX)

Table S4 **Average Scores for Equine Databases.** The average scores for the equine databases indicate a high degree of sequence alignment with the sequences that matched. [HspScore (high-scoring segment pair)- measures degree of local alignments with no gaps. Higher scores indicate better alignment. BitScore- statistical accounting of the raw alignment score which is the sum of the substitution and gap scores. Higher scores indicate better alignment. Average hit length- the length of the sequences that align.]
(DOCX)

Table S5 **Number of significant genes for each analysis.** The number of significant genes for each analyse (with and without duplicate removal) was determined. An ANOVA with interactions (p<0.05) was used to determine significance.
(DOCX)

Table S6 **Canonical pathways for all analyses.** All significant canonical pathways for all analyses are listed. The * denotes pathways involved with the nervous system (11), while the ∧ denotes pathways involved with the immunological response (4).
(DOCX)

Table S7 **Functions for all analyses.** The number of transcripts for significant functions for all analyses are listed. The * denotes functions involved with the nervous system (6) while the ∧ denotes functions involved with the immunological system (11).
(DOCX)

Table S8 **Transcripts for all analyses mapped to immunological CPs.** This table shows the number of transcripts that mapped to each pathway for all analyses. The majority of the transcripts demonstrated a decrease in expression values. Transcripts were included if they demonstrated a fold change >1 or <−1.
(DOCX)

Table S9 **Specific clinical signs of horses by group analyzed by CNS microarray.** Horses were graded as no increase in clinical signs, a moderate to mild increase in clinical signs, and a severe increase in clinical signs for all studies. Clinical signs observed included changes in mentation, paresis, ataxia, and muscle fasciculations. The nonvaccinated group exposed to West Nile virus demonstrated the most severe increase in severity of clinical signs, while the vaccinated/exposed group and normal controls did not show any changes in clinical signs.
(DOCX)
