# Supplementary material for: Gene Expression Analysis in the Thalamus and Cerebrum of Horses Experimentally Infected with West Nile Virus
Source: PLoS One. 2011 Oct 4;6(10):e24371. doi: 10.1371/journal.pone.0024371 (PMC3186766; doi:10.1371/journal.pone.0024371)
Supplement: Methods S1 — Supplemental methods information, including information on RNA extraction and quality assessment, the construction and normalization of the cDNA library, production of samples for analysis on the array, hybridization and scanning of the arrays, and microarray validation. (DOCX) [file pone.0024371.s001.docx]

**Methods S1**

RNA Extraction and Quality Analysis for Pyrosequencing

Total RNA was extracted from the tissues from 2 horses for each of the 3 groups for pyrosequencing (8 tissues were collected from each horse and included cerebrum, cerebellum, thalamus, midbrain (rostral and caudal colliculus, tectum, and tegmentum), hindbrain (pons and medulla oblongata), cervical spinal cord, lumbar spinal cord, and spleen for a total of 48 tissues). A 30 mg piece of tissue was weighed out for each sample on dry ice. The tissues were homogenized using manual disruption and placed in 1 mL of guanidium thiocyanate (Trizol ®, Invitrogen, Carlsbad, CA). The samples were vortexed and allowed to remain at room temperature for 5 minutes to allow complete dissociation of the nucleoprotein complexes. Two hundred μL of molecular grade chloroform (Fisher Scientific ®) was added to each sample. The samples were placed at room temperature for 2 minutes, then centrifuged at 12,000 x *g* at 4^o^C for 15 minutes. The chloroform and centrifugation step were repeated to ensure complete removal of the lipids. A 0.5 mL aliquot of isopropanol alcohol was added to each sample and incubated at room temperature for 5 minutes. The samples were centrifuged at 12,000 x *g* at 4^o^C for 10 minutes to precipitate the RNA. One mL of 75% ethanol was added to each pellet, mixed, and centrifuged at 7,500 x *g*, for 5 minutes at 4^o^C. The ethanol was poured off and the pellets air dried for 5 minutes. RNAsecure® (Ambion, Austin, TX), diluted to a 1X concentration, was heated on a heat block at 60^o^C for 5 minutes and 75 μL added to each pellet to inactivate any residual RNases. The pellets were incubated at 60^o^C for 10 minutes in RNAsecure and cooled to room temperature. For DNase treatment, 7.5 μL of 10X DNase buffer and 1 μL of rDNase (Ambion®, Austin, TX) was added to each sample. Samples were incubated at 37^o^C for 1 hour. After incubation, 7.5 μL of DNase inactivating reagent® (Ambion, Austin, TX) were added to each sample and the samples were incubated at room temperature for 2 minutes. The samples were centrifuged at 10,000 rpm for 2 minutes, removed from the inactivating reagent, and placed at -80^o^C until quality assessment.

RNA quality assessment was performed using a microfluidics platform. One μL of each RNA sample was placed on a nano-drop machine (ND-1000, Nanodrop Technologies, Wilmington, DE). The concentration and 260:280 ratio of each sample was assessed. One μL of each RNA sample was then run on the Agilent 2100 Bio-analyzer (Santa Clara, CA) to assess the degree of RNA degradation. Briefly, the sample was incubated with fluorescent dyes, run on a gel, and excited with a laser to generate an electropherogram. From this electropherogram, the ratio of the 28S and 18S ribosomal peaks was obtained and software extraction performed. A RNA integrity number (RIN) was generated with high values (6-10) corresponding with tall 28S and 18S rRNA peaks and a low baseline, indicating minimal degradation of the RNA and a high quality sample. Low values (1-5) corresponding with a shift in the height of the 28S and 18S rRNA peaks and an increasing baseline, indicating a large amount of RNA degradation and low quality samples. Only samples with a RIN >6 were used for the study.

First Strand cDNA Synthesis and Amplification- cDNA Library Construction

The RNA isolated from the tissue samples from each horse were pooled to create 5 samples total. The RNA was converted to full-length, double-stranded cDNA using the SMART PCR cDNA synthesis kit, the Advantage® 2 PCR kit, and the PowerScript Reverse Transcriptase (Clontech, Mountainview, CA). To create the first strand of cDNA, 1 μg of RNA sample was mixed with 1μL 3’SMART CDS Primer II A, 1μL SMART II A Oligonucleotide mix and DI Water for a total volume of 5 μL. The contents of the tube were mixed, centrifuged briefly, and incubated in a thermocycler at 70^o^C for 2 minutes. Two μLs 5X first-strand buffer, 1μL DTT, 1μL dNTP mix, and 1μL MMLV Reverse Transcriptase were added to each tube. The tubes were mixed, centrifuged briefly, and incubated at 42^o^C for 1 hr in an air incubator. Tris EDTA buffer (10mM Tris, 1mM EDTA) at a volume of 40μL was added to each tube and the tubes were heated at 72^o^C for 7 minutes. One μL of cDNA was added to 9 μL of deionized water to amplify the cDNA. Seventy four μL DI water, 10μL 10X Advantage 2 PCR Buffer, 2μL 50X dNTP, 2μL 5’PCR Primer II A, and 2μL 50X Advantage 2 Polymerase Mix was added for a total volume of 90μL. The tubes were vortexed and centrifuged. The tubes were held at 95°C for 1 minute, then cycled 17 times with the following parameters: 95°C for 15 seconds, 65°C for 30 seconds, and 68°C for 6 minutes. The amplified cDNA was then analyzed using a micro-fluidics platform to determine concentration and purity.

The cDNA was purified using a proprietary PCR purification kit (QIAquick PCR Purification Kit®, Qiagen, Valencia, CA). Proprietary binding buffer (PB1) at a volume of 450 μL was added to the 90 μL cDNA sample from SMART PCR cDNA Synthesis kit and placed in a QIAquick spin column. The samples were centrifuged at 13,000 rpm for 30–60 s. The flow-through was discarded, and 0.75 mL of proprietary cleansing buffer (PE) was added to the columns and centrifuged at 13,000 rpm for 60 s. The flow-through was discarded and the column centrifuged 13,000 rpm for an additional 1 minute. The DNA was then eluted by adding 50 μL water (pH 7.0–8.5) to the center of the membrane and centrifuging the column at 13,000 rpm for 1 min. A micro-fluidics platform was used to check the concentration and purity of the sample.

Normalization of the cDNA Library

The purified cDNA sample was normalized to ensure equal expression of all transcripts (cDNA Normalization Trimmer Kit®, Evrogen, Moscow, Russia). All tubes containing the cDNA samples (5 total) were combined and 1000 ng of purified cDNA was placed into a 2 mL Sarstedt tube. Three Molar sodium acetate (0.1 volumes), pH 4.8 was added, then 2.5 volumes of 98% ethanol was added and the tube vortexed. The sample was centrifuged for 15 min at 13,000 rpm and the supernatant removed. One hundred μL of 80% ethanol was laid over the pellet. The tubes were centrifuged at 13,000 rpm for 5 minutes. The supernatant was removed and the ethanol wash repeated. The pellet was air dried for 15 min at room temperature, then dissolved in sterile water to the final cDNA concentration of 100-150 ng/μL.

Eight μLs of ds cDNA were combined with 4 μL 4X hybridization buffer and 4 μL sterile water to begin hybridization. The contents were mixed and 4 μL aliquoted into each of four tubes. The tubes were centrifuged at 14,000 rpm for 2 min, incubated in a thermal cycler at 98oC for 2 min, and incubated at 68oC for 5 hours. Proprietary duplex specific endonuclease (DSN) treatment was performed. Two tubes were created- one with a 1:1 ratio of DSN storage buffer to 1 μL of DSN solution, the other with a 3:1 ratio of 3 μL of DSN storage buffer to 1 μL of DSN solution. The DSN master buffer was preheated at 68oC and 5 μL was added to each tube containing hybridized cDNA. The tubes were centrifuged and incubated at 68oC for 10 min. The DSN enzyme was added to each tube as specified in Table 3-2. DSN functions to degrade double-stranded cDNA which should only be present after denaturing and reannealing with the abundant transcripts. The tubes were incubated in the thermal cycler at 68^o^C for 25 min. Ten μLs of DSN stop solution was added to each tube, and the tubes were mixed and centrifuged. The tubes were incubated in the thermal cycler at 68oC for 5 min, then placed on ice. Twenty μLs of sterile water was added to each tube.

The amplification steps of the normalized cDNA were performed. A master mix was prepared ( 40.5 μL Sterile water, 5 μL 10X PCR Buffer, 1 μL 50X dNTP mix, 1.5 μL Evrogen PCR primer M1, and 1 μL 50X Polymerase Mix) and 49 μL added to 1 μL of each diluted cDNA sample (from DSN treatment, see above). The tubes were mixed and centrifuged briefly. Amplification was performed by cycling 18 times at 95oC for 7s, 66oC for 30 s, and 72oC for 6 min. The samples were run on a gel and the wells containing normalized samples were combined. A second amplification step was then performed. Two μLs of normalized cDNA was combined with 20 μL of sterile water. The tube was mixed and centrifuged briefly. For a control, 2 μL of control cDNA was aliquoted into another tube and combined with 20 μL of sterile water. The tube was mixed and centrifuged briefly. Two μL of each of these diluted samples (control and normalized) were then mixed with a master mix consisting of 80 μL Sterile water, 10μL 10X PCR Buffer, 2 μL 50X dNTP mix, 4 μL Evrogen PCR primer M2, and 2 μL 50X Polymerase Mix. PCR was performed on both tubes cycling 12 times at 95oC for 7s, 64^o^C for 10s, and 72oC for 6 min. The concentration and purity of the normalized equine WNV library was determined using a micro-fluidics platform and run on a gel to assess concentration and purity.

RNA Extraction for Microarray Analysis

Total RNA was extracted from 6 horses from each of the 3 groups for analysis on the array (thalamus and cerebrum of each for a total of 36 samples). A 30 mg piece of tissue was weighed out for each sample on dry ice. The tissues were homogenized using manual disruption and placed in 1 mL of guanidium thiocyanate (Trizol®, Invitrogen, Carlsbad, CA). The samples were vortexed and allowed to remain at room temperature (RT) for 5 minutes to allow complete dissociation of the nucleoprotein complexes. Two hundred μL of molecular grade chloroform (Thermo Fisher Scientific ®, Waltham, MA) was added to each sample. The samples were placed at room temperature for 2 minutes, then centrifuged at 12,000 x *g* at 4^o^C for 15 minutes. The chloroform and centrifugation steps were repeated to ensure complete removal of the lipids. A 0.5 mL aliquot of isopropanol alcohol was added to each sample and incubated at room temperature for 5 minutes. The samples were centrifuged at 12,000 x *g* at 4^o^C for 10 minutes to precipitate the RNA. One mL of 75% ethanol was added to each pellet, mixed, and repelleted using centrifugation (7,500 x *g*, 4^o^C, 5 minutes). The ethanol was poured off and the pellets air dried for 5 minutes. RNAsecure (Ambion ®, Austin, TX) diluted to a 1X concentration was heated on a heat block at 60^o^C for 5 minutes and 75 μL was added to each pellet to inactivate any residual RNases. The pellets were incubated at 60^o^C for 10 minutes in RNAsecure and cooled to room temperature. For DNase treatment, 7.5 μL of 10X DNase buffer and 1 μL of rDNase (Ambion®, Austin, TX) was added to each sample. Samples were incubated at 37^o^C for 1 hour. After incubation, 7.5 μL of DNase inactivating reagent (Ambion®, Austin, TX) were added to each sample and the samples were incubated at room temperature for 2 minutes. The samples were centrifuged at 10,000 rpm for 2 minutes, removed from the inactivating reagent, and placed at -80^o^C until quality assessment. One μL of each RNA sample was placed on a nano-drop machine (ND-1000, Nanodrop Technologies, Wilmington, DE). The concentration and 260:280 ratio of each sample was assessed using spectrophotometric technology. Only samples with a RIN > 6 and a 260:280 ratio > 1.8 were used.

cDNA Creation and Dye Labeling

Dye-labeled cDNA was created using Cy3 dye (One-Color Microarray-Based Gene Expression Analysis kit, Agilent Technologies). The first strand cDNA was created using 3000 ng of RNA in 9 μL or less was aliquoted into individual tubes. 2.5 μL of T7 promoter primer was added to each tube and the tubes were incubated at 65^o^C for 10 minutes, then placed on ice for 5 minutes. The proprietary master mix (Agilent Technologies) was added to each tube (8.5 μL) consisting of 4 μL of 5X first strand buffer (pre-warmed at 80^o^C), 2 μL of 0.1M DTT, 1 μL of 10mM dNTP mix, 1 μL MMLV, and 0.5 μL RNAse inhibitor. The tubes were incubated at 40^o^C for 2 hours, heated to 65^o^C for 15 minutes, and incubated on ice for 5 minutes. The amplification mixture for dye incorporation consisting of 30 μL of master mix (15.3mL of nuclease-free water, 20 μL 4X transcription buffer, 6 μL 0.1M DTT, 8 μL NTP, 6.4 μL 50% PEG pre-warmed at 40^o^C for 1 minute, 0.5 μL RNAse OUT, 0.6 μL inorganic pyrophosphate, 0.8 μL T7 RNA polymerase, and 2.4 μL cyanine 3-CTP dye) was added and each tube was incubated at 40^o^C for 2 hours.

The dye-labeled cDNA was then extracted using a propriety kit (RNeasy®,Qiagen, Valencia, CA). The samples were brought to a total volume of 100 μL by adding 20 μL of nuclease free water and 350 μL of the kit buffer (RLT) was added to each sample and thoroughly mixed with a pipette. Ethanol (100%, 250 μL) was added to each sample and mixed thoroughly with a pipette. Seven-hundred μLs of each sample were transferred to the kit column,and the columns were centrifuged at 13,000 rpm for 30 seconds at 4^o^C. The columns were transferred to a new collection tube and 500 μL of buffer (RPE) was added to each column. The columns were centrifuged for 60 seconds at 13,000 rpm at 4^o^C, and the eluant discarded. The buffer RPE and centrifugation steps were repeated. The columns were transferred to a new collection tube and air dried for 2 minutes. To each column 30 μL of RNase-free water was added. The columns were incubated for 1 minute at room temperature and were then centrifuged for 30 seconds at 13,000 rpm at 40^o^C and this step was repeated with the same sample.

The specific activity and yield of the samples were determined using a microfluidics platform (Nano-drop Technologies, Thermo Scientific). Both the concentration and the incorporation of the dye were measured. The formula Specific Activity = [(Concentration of Cy3)/(Concentration of cRNA)] * 1000 = pmol Cy3 per μg cRNA was used to determine whether the sample would be used for hybridization to the array. Only samples with a specific activity > 8 were used.

Hybridization and Scanning of Arrays

Hybridization to the microarrays was performed according to the manufacturer’s protocol. Individual, non-pooled cDNA samples were hybridized to the arrays. Briefly, the prioprietary blocking agent was prepared to a 10X concentration and incubated at 37^o^C for 5 minutes. Individual tubes were prepared combining 1.65 mg of dye-labeled cDNA, 11 mL of 10X blocking agent, nuclease-free water, and 2.2 mL of the proprietary 25X fragmentation buffer for a final volume of 55 mL. The tubes were incubated at 60^o^C for 30 minutes and then placed on ice for 1 minute. Fifty mL of the proprietary 2X hybridization buffer was added to each tube. The samples were mixed, centrifuged at 13,000 rpm for 1 minute at 25^o^C, and placed on ice. The gaskets were placed into the chambers and 100 mL of sample added to each chamber. The array slides were placed on top of each gasket. The chambers were closed in the hybridization oven and rotated at 10 rpm for 17 hours at 65^o^C.

After the hybridization, the arrays were disassembled in wash buffer 1. The slides were washed for 1 minute in proprietary wash buffers at 37^o^C. The excess liquid was dried off and the slides washed for 30 seconds in the proprietary stabilization and drying solution. The slides were scanned and data collected using the proprietary software (Feature Extraction®, Agilent).

Microarray Validation

For the purposes of the initial validation of the utility of this microarray, several highly significant genes (six) were selected to 1) verify the accuracy of the probe hybridization, and 2) verify the accuracy of the relative expression values detected by the probe. To verify the relative expression values, only transcripts that were significantly upregulated or downregulated (p<0.05, fold change >2, <-2) in the exposure analysis were picked for analysis. A total of six transcripts were targeted to be used as primer sets in the validation experiment and included 2’5’ oligoadenylate synthetase (2,5 OAS), complement component 1 (CC1), TNFα receptor ligand (TNFR), interleukin-6 (IL-6) , DEAD Box 60 (DB60), and defensin β4 ( DB4), with β -actin (ACT) as the endogenous control. Two sets of primers were designed using primer design software (ABI Primer Express version 3.0, Applied Biosystems). The primers are available upon request. The first set of primers was designed to amplify a larger segment of the gene. Conventional PCR was performed using a proprietary master mix (Readymix Taq PCR Mastermix with MgCl_2_, Sigma-Aldrich, St.Louis, MO). For each primer reaction, 25μL of the reagent mix, 1 μL each of forward and reverse primer (10mM), 5 μL of sample, and 18 μL of water were added to each respective PCR tube. The samples were held at 94^o^C for 2 minutes, then cycled 25 times at 94^o^C for 1 minute, 50^o^C for 2 minutes, and 72^o^C for 3 minutes. The samples were then held at 72^o^C for 5 minutes and cooled at 4^o^C. The reactions were run in triplicate for each set of primers.

The three tubes from each reaction were combined and purified using a PCR purification kit (QIAquick® PCR purification kit, Qiagen, Valencia, CA). Briefly, 5 volumes of the kit binding buffer (PB1) was added. The samples were mixed and placed on the kit column. The tubes were centrifuged at 13,000 rpm (>10,000g) for 60 seconds. The eluant was discarded and 750 μL of wash buffer (PE) was added. The columns were centrifuged at 13,000 rpm for 60 seconds, the eluant discarded, and the columns centrifuged again at 13,000 rpm for 60 seconds. Thirty μLs of water was added to each column membrane and the columns centrifuged again at 13,000 rpm for 60 seconds. The concentration and purity of the samples were determined using a microfluidics platform. The reactions were resolved utilizing a 0.9% agarose gel and imaged under standard UV conditions. If a band(s) was visualized, the samples were submitted to the UF Interdisciplinary Centers for Biotechnology Research for Sanger sequencing.

Sequencing results were checked against expected gene sequences. Once the correct sequence was validated, amplified samples were run under the thermocycling conditions listed above using a second set of nested primers. The presence of a band of the correct length was verified on a 0.9% agarose gel. For each target, a standard curve was generated using 5 two-fold dilutions and triplicate wells. The slope of the reaction and the R square was calculated via the proprietary software (ABI 7900, Applied Biosystems). The primer efficiency was checked using the equation efficiency = 10^(-1/slope). These primers were then used in real time, relative quantitation PCR in a SYBR green assay (Fast SYBR Green Master Mix, Applied Biosystems) to validate the findings of the level of expression demonstrated via array. Using proprietary conditions, 10 μL of Fast SYBR Green Master Mix, a variable amount of each primer dependent on reaction efficiency, 3000 ng of cDNA, and water up to a volume of 20 μL were added to each well with replicates of three wells performed on each sample. The plate was centrifuged and the real time PCR reactions (7500 Fast Real-Time PCR System, Applied Biosystems) were performed using the reaction parameters consisting of a hold at 95^o^C for 20 seconds, followed by 40 cycles consisting of a 40 cycle reaction at 95^o^C for 3 seconds and a 40 cycle reaction at 60^o^C for 30 seconds. Relative quantitation analysis was performed using the proprietary software for calculation of the comparative Ct method (Applied Biosystems software for the 7500 Fast machine) wherein 2^-∆∆Ct^ is used for the comparison of relative quantitation between the thalamus of vaccinated/exposed horses and nonvaccinated/exposed horses.

To verify the accuracy of the probe hybridizations, the probe sequences were BLASTed against the equine genome (Fisher Cluster, UF ICBR, Gainesville, FL). Only sequences with e-values <10-4 were generated. Sequences were checked for percent identity and sequence alignment.
