# Supplementary figures and images for: Gene Expression Analysis in the Thalamus and Cerebrum of Horses Experimentally Infected with West Nile Virus
Source: PLoS One. 2011 Oct 4;6(10):e24371. doi: 10.1371/journal.pone.0024371 (PMC3186766; doi:10.1371/journal.pone.0024371)

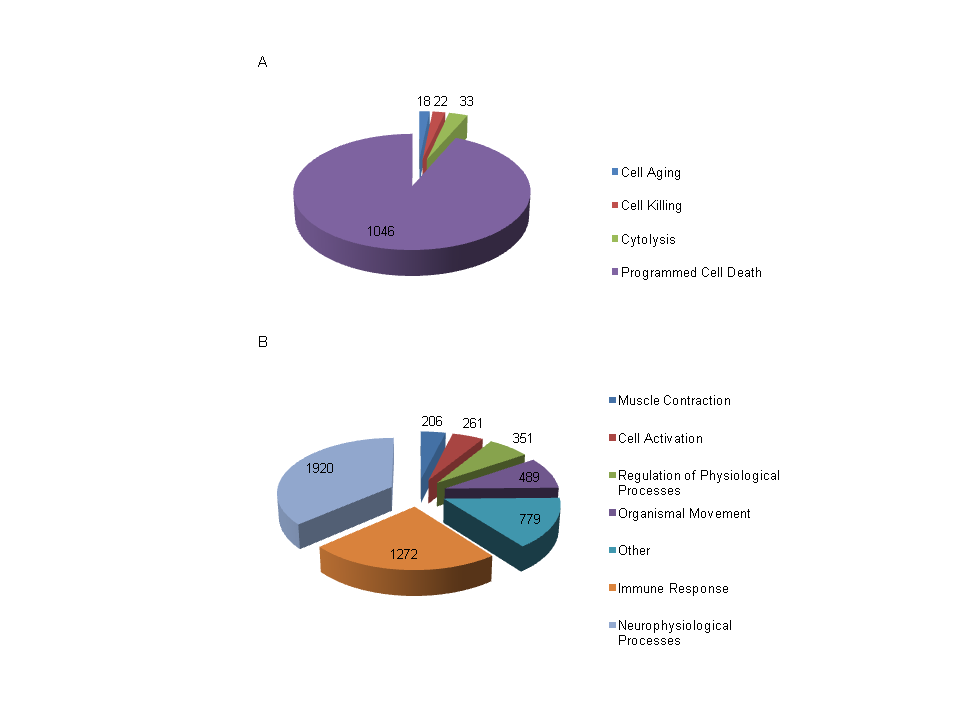

Supplement: Figure S1 — Gene ontology classification of physiological processes. These categories were included under biological process. The majority of genes were involved with neurophysiological processes (1,920) and the immune response (1,272). (TIF) [file pone.0024371.s002.tif]

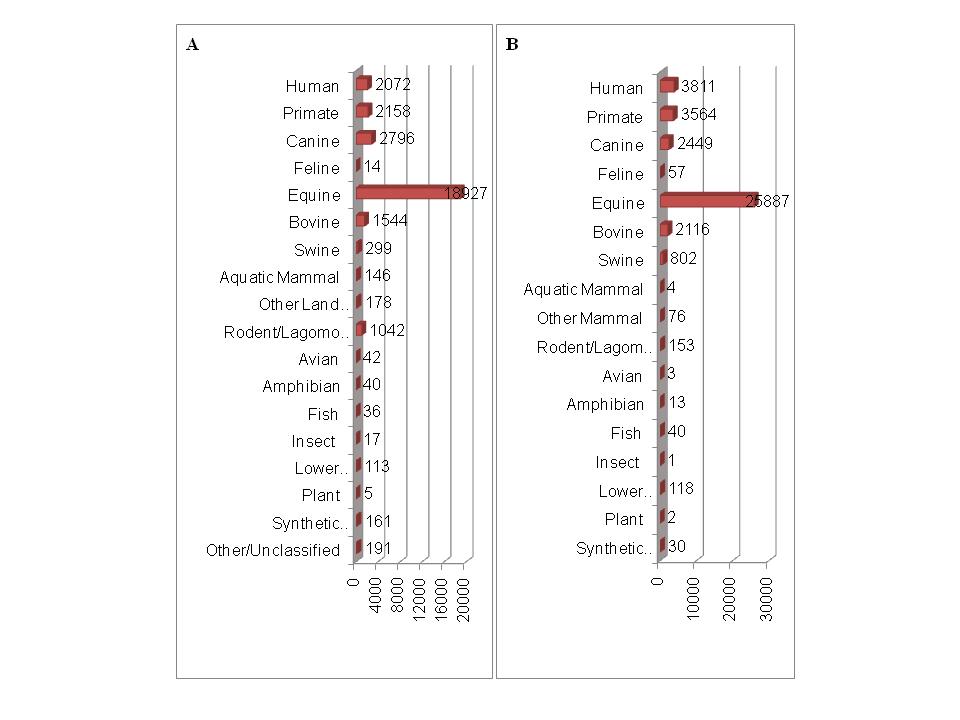

Supplement: Figure S2 — Sequence count by species group for the NCBI NR/NT databases. A.) Sequence count for the NR database. The majority of sequences mapped to the horse, with other prominent groups including the human, primate, canine, and bovine. B.) Sequence count for the NT database. The majority of sequences in this database also mapped to the horse, with other prominent groups including the human, primate, canine, and bovine. (TIF) [file pone.0024371.s003.tif]

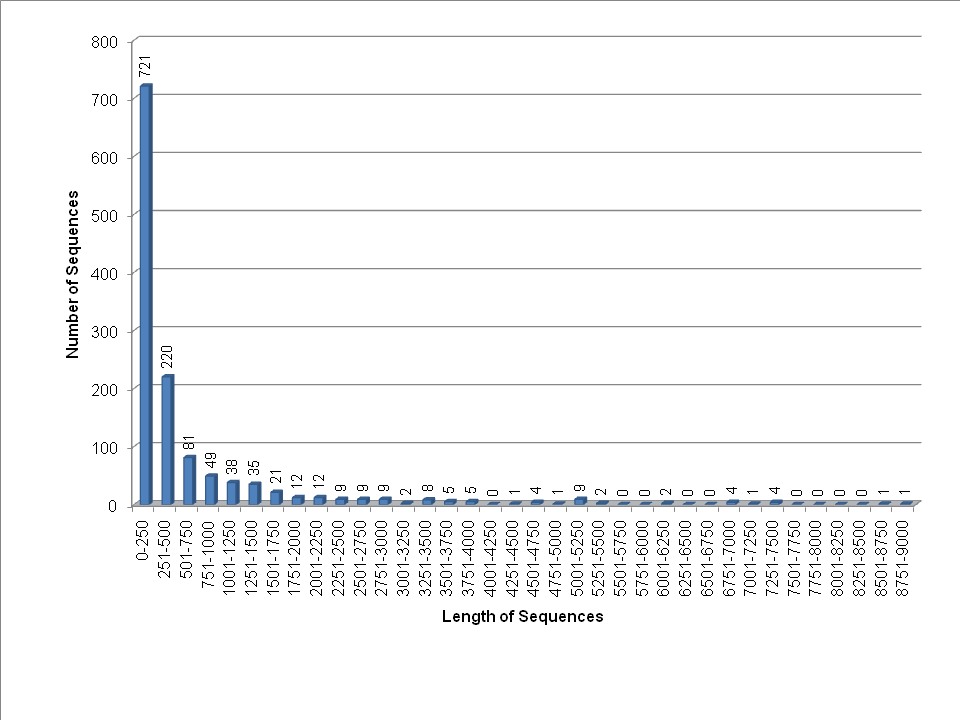

Supplement: Figure S3 — Average length of novel sequences. The majority of sequences annotated were less than 1000 base-pairs, with an average length of 595 base-pairs. (TIF) [file pone.0024371.s004.tif]

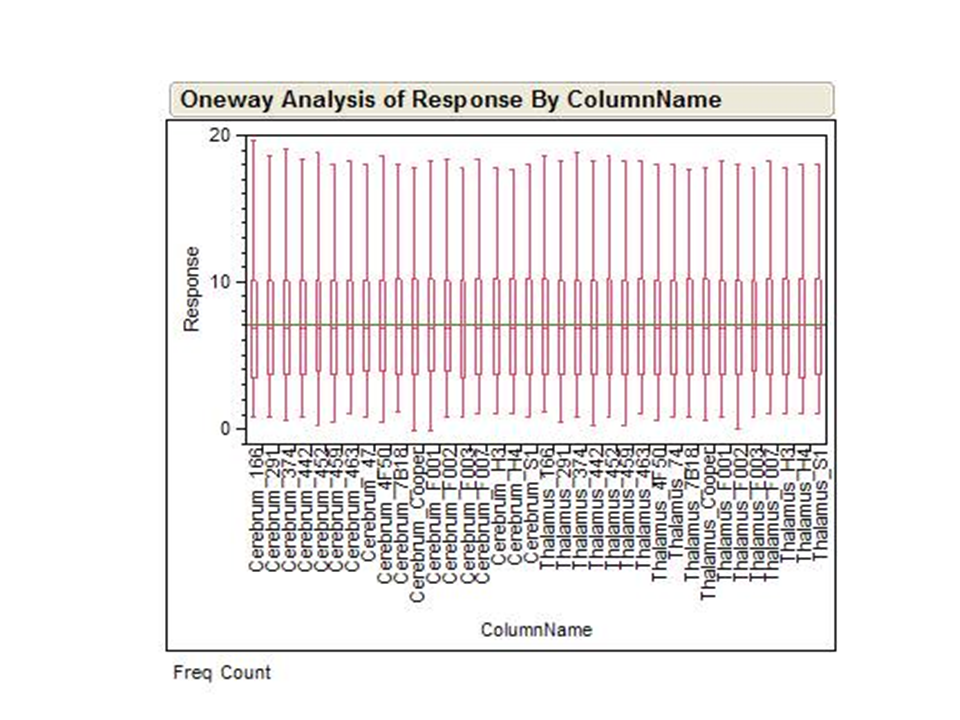

Supplement: Figure S4 — Box plots for Loess normalization. The green line indicates the mean of all arrays after normalization, while the red boxes indicate the range of response, the red lines in the boxes the median of each array, and the extended red lines standard deviations. All arrays normalized correctly. (TIF) [file pone.0024371.s005.tif]

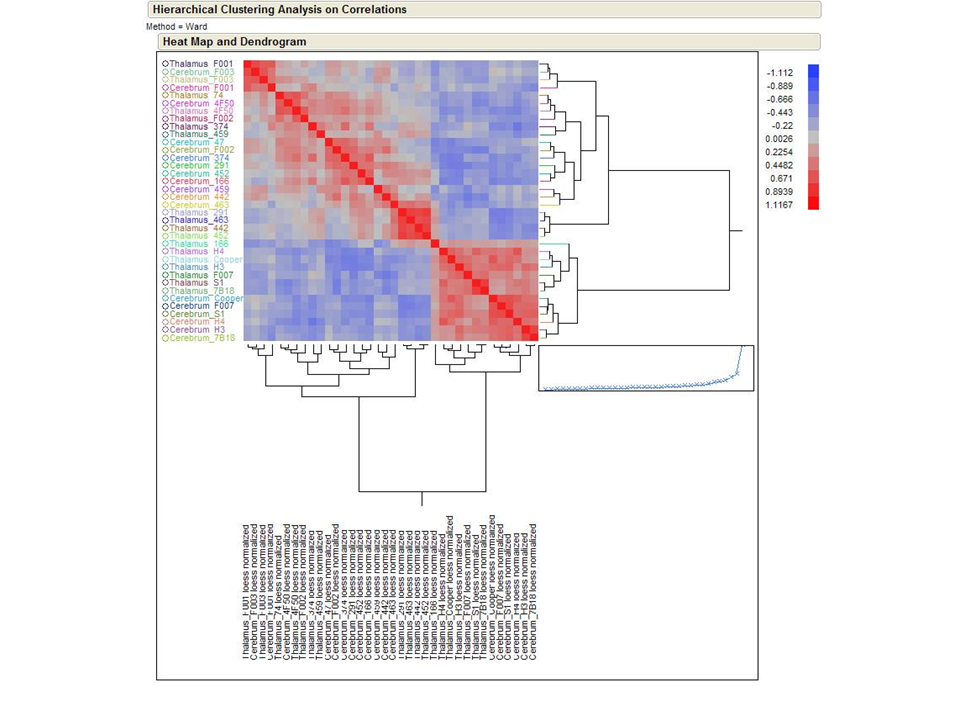

Supplement: Figure S5 — Heat Map and Dendrogram of All Arrays Demonstrating Similarity in Gene Expression. Dark red indicates a high degree of similarity, while blue indicates a low degree of similarity. (TIF) [file pone.0024371.s006.tif]

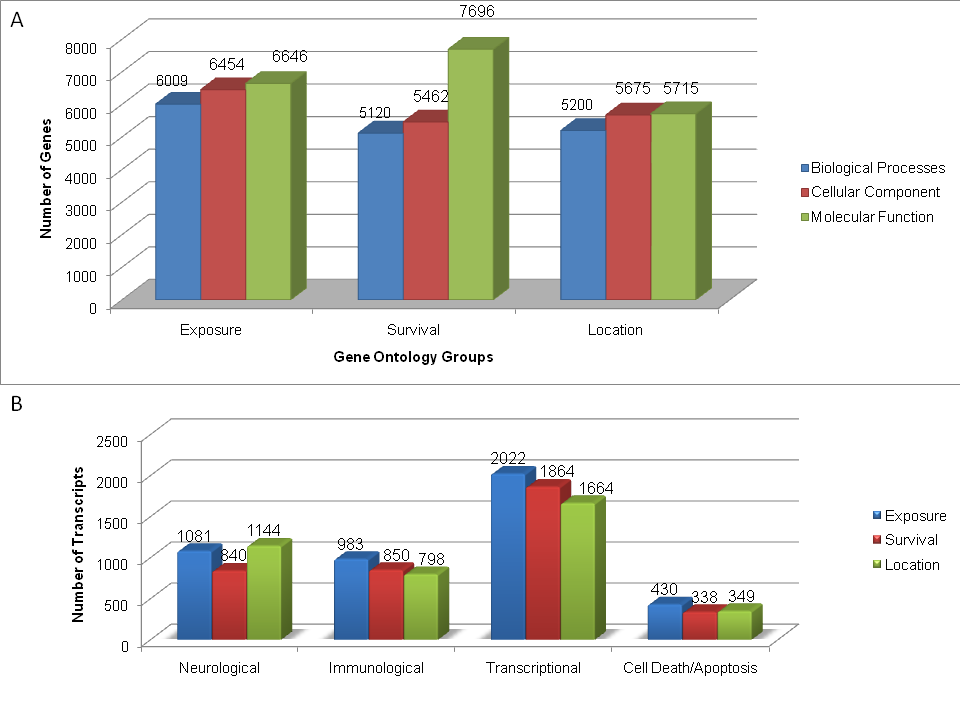

Supplement: Figure S6 — Number of Genes that Mapped to GO Categories for All Analyses and Distribution of Genes Among GO Categories. A. The distribution of genes is relatively even, with slightly more genes overall in the exposure analysis compared to the nonsurvival and location analysis. B. Most genes for all analyses were classified under transcriptional categories, with neurological categories containing the second highest number of genes. For the purposes of this study, ‘exposure’ represents the difference in gene expression between the nonvaccinated/exposed-normal, ‘survival’ represents the difference in gene expression between the nonvaccinated/exposed-vaccinated/exposed, and ‘location’ represents the difference in gene expression between the thalamus and cerebrum of the nonvaccinated/exposed. (TIF) [file pone.0024371.s007.tif]

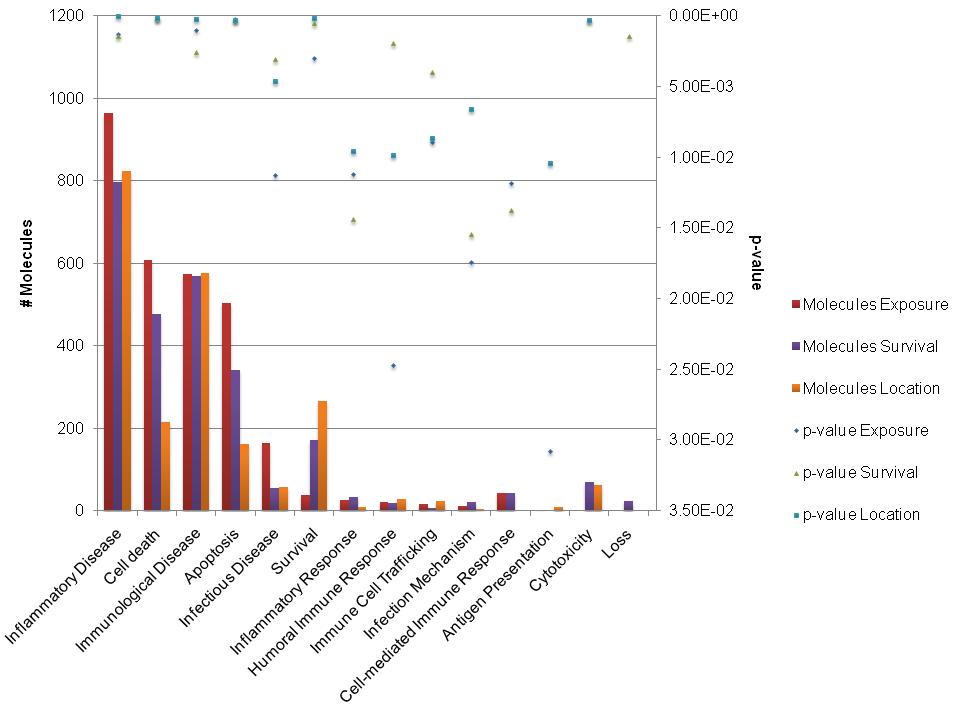

Supplement: Figure S7 — Immunological and cell death/apoptosis functions for all analyses. The majority of all transcripts mapped to the exposure analysis. Both innate and adaptive immune categories are present, as well as both cell death and apoptosis. For the purposes of this study, ‘exposure’ represents the difference in gene expression between the nonvaccinated/exposed-normal, ‘survival’ represents the difference in gene expression between the nonvaccinated/exposed-vaccinated/exposed, and ‘location’ represents the difference in gene expression between the thalamus and cerebrum of the nonvaccinated/exposed. (TIF) [file pone.0024371.s008.tif]
