# Supplementary material for: Gene Expression Analysis in the Thalamus and Cerebrum of Horses Experimentally Infected with West Nile Virus
Source: PLoS One. 2011 Oct 4;6(10):e24371. doi: 10.1371/journal.pone.0024371 (PMC3186766; doi:10.1371/journal.pone.0024371)
Supplement: Table S1 — Probe groups for inclusion on the microarray. Probes were included on the array once in the ‘plus’ (5′–3′) orientation (Annotated). Probes were individually selected to be included twice on the array (Important analyse) and included sequences involved in neurological, immunological, and transcriptional processes, as well as cell death. Probes that were determined to be correctly oriented in the ‘minus’ (3′–5′) direction were included in Annotated_minus. Unannotated probes and probes recovered from the EqCab2 genome sequencing project were also included. 250 Agilent controls were incorporated. (DOCX) [file pone.0024371.s009.docx]

**Table S1 Probe groups for inclusion on the microarray**

| Probe group | # of probes | Replicates |
| --- | --- | --- |
| Important | 3,883 | 1 |
| Annotated | 28,600 | 1 |
| Annotated_minus | 1,567 | 1 |
| Unannotated | 3,906 | 1 |
| Recovered_genome | 5,444 | 1 |
| Control | 250 | 1 |
