# Supplementary material for: Gene Expression Analysis in the Thalamus and Cerebrum of Horses Experimentally Infected with West Nile Virus
Source: PLoS One. 2011 Oct 4;6(10):e24371. doi: 10.1371/journal.pone.0024371 (PMC3186766; doi:10.1371/journal.pone.0024371)
Supplement: Table S2 — Average Nucleic Acid Quality Data for All Samples. Quality assessment was performed on all RNA samples used to create the cDNA library. The quality of the RNA was checked using spectrophotometric technology (ND-1000, Nanodrop Technologies, Wilmington, DE) and a microfluidics platform (Agilent 2100 Bio-analyzer, Santa Clara, CA). Only samples with a RIN>6 and a 260∶280 ratio >1.80 were used in the experiment. Quality assessment was also performed on the cDNA libraries including the 260∶280 ratio (>1.80) and concentration measurements. (DOCX) [file pone.0024371.s010.docx]

**Table S2. Average Nucleic Acid Quality Data for All Samples**

| Sample | Concentration (ng/uL) | RIN | 260:280 Ratio |
| --- | --- | --- | --- |
| Naïve + Non-exposed RNA | 411.63 (152-652.53) | 7.1 (6-7.8) | 1.96 (1.8-2.08) |
| Non-naïve + Exposed RNA | 829.55 (122-1551) | 7.4 (6.7-8.4) | 1.99 (1.87-2.06) |
| Naïve + Exposed RNA | 439.02 (182-968) | 7.6 (6.7-8.4) | 1.95 (1.75-2.04) |
| Naïve + Non-exposed cDNA Library | 512.24 | N/A | 1.82 |
| Non-naïve + Exposed cDNA Library 1 | 474.31 | N/A | 1.84 |
| Non-naïve + Exposed cDNA Library 2 | 644.69 | N/A | 1.84 |
| Naïve + Exposed cDNA Library 1 | 452.86 | N/A | 1.85 |
| Naïve + Exposed cDNA Library 2 | 640.41 | N/A | 1.83 |
