# Supplementary material for: Gene Expression Analysis in the Thalamus and Cerebrum of Horses Experimentally Infected with West Nile Virus
Source: PLoS One. 2011 Oct 4;6(10):e24371. doi: 10.1371/journal.pone.0024371 (PMC3186766; doi:10.1371/journal.pone.0024371)
Supplement: Table S3 — Data from 454 Sequencing Runs Newbler Assembler. Newbler assembly software was first used to assemble the sequences. 514,462 sequences were assembled from a total of 49,857,586 bases after linker contamination was removed. Sequences that could be linked from beginning to end were classified as ‘fully assembled reads’, sequences that were shown to have some association were classified as ‘partially assembled reads’, standalone sequences coding once for areas of individual genes were classified as ‘singletons’, standalone sequences coding for areas of individual genes at a frequency of greater than 5% were classified as ‘repeats’, and standalone sequences coding for areas of individual genes at a frequency of less than 5% were classified as ‘outliers’. From this data, 16,895 contigs (sets of overlapping DNA sequences) composed of 4,720,747 bases were assembled. (DOCX) [file pone.0024371.s011.docx]

**Table S3. Data from 454 Sequencing Runs Newbler Assembler**

| Reads | |
| --- | --- |
| Total # of reads | 826,176 |
| Total # of clean reads | 514,412 (62.3%) |
| Total # of bases | 95,486,897 |
| Total # of clean bases | 49,857,586 (52.2%) |
| # of fully assembled reads | 70,828 |
| # of partially assembled reads | 64,823 |
| # of singletons | 276,760 |
| # of repeat reads | 93,504 |
| # of outlier reads | 8,497 |
| All Contigs | |
| # of all contigs | 16,895 |
| # of bases covered | 4,720,747 |
| Avg. contig size | 279.4 |
| Min. contig size | 93 |
| Max contig size | 2,827 |
| Large Contigs | |
| # of large contigs | 1,902 |
| # of bases covered | 1,557,286 |
| Avg. large contig size | 818 |
| Min. large contig size | 500 |
| Max. large contig size | 2,827 |
