# Supplementary material for: Gene Expression Analysis in the Thalamus and Cerebrum of Horses Experimentally Infected with West Nile Virus
Source: PLoS One. 2011 Oct 4;6(10):e24371. doi: 10.1371/journal.pone.0024371 (PMC3186766; doi:10.1371/journal.pone.0024371)
Supplement: Table S4 — Average Scores for Equine Databases. The average scores for the equine databases indicate a high degree of sequence alignment with the sequences that matched. [HspScore (high-scoring segment pair)- measures degree of local alignments with no gaps. Higher scores indicate better alignment. BitScore- statistical accounting of the raw alignment score which is the sum of the substitution and gap scores. Higher scores indicate better alignment. Average hit length- the length of the sequences that align.] (DOCX) [file pone.0024371.s012.docx]

**Table S4. Average Scores for Equine Databases**

|  | EqCab2 Chromosomes | EqCab2 Predicted Genes | EqCab2 ab initio Predicted Genes by GeneScan |
| --- | --- | --- | --- |
| Average HspScore | 492.3956 (17-11,585) | 1326.042 (14-23,780) | 643.1346 (15-11,553) |
| Average BitScore | 976.5968 (34.193-22,966.1) | 2629.181 (508-47,141) | 1275.415 (30.2282-22,902.7) |
| Average Hit Length | 501.524 | 1328.69 | 647.97 |
| # of Sequences with % Positive Identity >95% | 40,145/40,973 (97.98%) | 40,264/40,999 (98.21%) | 39,650/40,977 (96.76%) |
