# Supplementary material for: Gene Expression Analysis in the Thalamus and Cerebrum of Horses Experimentally Infected with West Nile Virus
Source: PLoS One. 2011 Oct 4;6(10):e24371. doi: 10.1371/journal.pone.0024371 (PMC3186766; doi:10.1371/journal.pone.0024371)
Supplement: Table S5 — Number of significant genes for each analysis. The number of significant genes for each analyse (with and without duplicate removal) was determined. An ANOVA with interactions (p<0.05) was used to determine significance. (DOCX) [file pone.0024371.s013.docx]

**Table S5. Number of significant genes for each analysis**

| **Analyses** | **Samples** | **Before duplicate removal** | **After duplicate removal** |
| --- | --- | --- | --- |
| Exposure Status | Nonvaccinate vs Control | 12,029 | 9,020 |
| Survival/Immune Status | Nonvaccinate vs Vaccinate | 9,978 | 7,395 |
| CNS Location | Nonvaccinate Cerebrum vs Thalamus | 10,555 | 7,649 |
