# Supplementary material for: Gene Expression Analysis in the Thalamus and Cerebrum of Horses Experimentally Infected with West Nile Virus
Source: PLoS One. 2011 Oct 4;6(10):e24371. doi: 10.1371/journal.pone.0024371 (PMC3186766; doi:10.1371/journal.pone.0024371)
Supplement: Table S6 — Canonical pathways for all analyses. All significant canonical pathways for all analyses are listed. The * denotes pathways involved with the nervous system (11), while the ∧ denotes pathways involved with the immunological response (4). (DOCX) [file pone.0024371.s014.docx]

**Table S6. Canonical pathways for all analyses**

|  | **Exposure status** | | **Survival/Immune status** | | **CNS location** | |
| --- | --- | --- | --- | --- | --- | --- |
| **Canonical Pathways** | **-log**  **(p-value)** | **Transcripts** | **-log**  **(p-value)** | **Transcripts** | **-log**  **(p-value)** | **Transcripts** |
| α-Adrenergic Signaling | 1.89E+00 | 11 | 2.21E+00 | 11 | 4.35E+00 | 14 |
| Amyotrophic Lateral Sclerosis Signaling* | 1.75E+00 | 12 | 1.64E+00 | 14 | 4.75E+00 | 15 |
| Antiproliferative Role of Somatostatin Receptor 2 |  |  |  |  | 3.49E+00 | 11 |
| Axonal Guidance Signaling* |  |  | 1.84E+00 | 29 |  |  |
| β-alanine Metabolism | 1.93E+00 | 8 |  |  |  |  |
| Breast Cancer Regulation by Stathmin1 |  |  |  |  | 4.95E+00 | 23 |
| Butanoate Metabolism | 1.55E+00 | 8 |  |  |  |  |
| Calcium Signaling |  |  | 2.68E+00 | 21 |  |  |
| Calcium Signaling |  |  |  |  | 3.45E+00 | 19 |
| cAMP-mediated Signaling | 1.71E+00 | 18 | 2.75E+00 | 18 | 4.04E+00 | 19 |
| Cardiac β-adrenergic Signaling | 1.99E+00 | 16 | 2.52E+00 | 16 | 5.05E+00 | 18 |
| Caveolar-mediated Endocytosis Signaling |  |  | 1.75E+00 | 8 |  |  |
| CDK5 Signaling* | 1.89E+00 | 11 | 1.76E+00 | 9 |  |  |
| Corticotropin Releasing Hormone Signaling | 1.55E+00 | 13 | 2.36E+00 | 13 |  |  |
| CREB Signaling in Neurons* | 2.41E+00 | 19 | 2.84E+00 | 21 | 6.23E+00 | 24 |
| CXCR4 Signaling^ |  |  |  |  | 3.71E+00 | 18 |
| Dopamine Receptor Signaling* | 2.41E+00 | 11 | 1.72E+00 | 8 |  |  |
| EGF Signaling |  |  |  |  | 3.92E+00 | 9 |
| Endothelin-1 Signaling | 2.07E+00 | 19 |  |  | 4.08E+00 | 20 |
| G Beta Gamma Signaling | 1.78E+00 | 11 | 2.11E+00 | 11 | 4.18E+00 | 14 |
| Glutamate Receptor Signaling* | 1.70E+00 | 9 | 5.02E+00 | 15 | 5.77E+00 | 13 |
| GNRH Signaling | 1.90E+00 | 15 |  |  |  |  |
| G-Protein Coupled Receptor Signaling |  |  | 3.40E+00 | 24 | 7.28E+00 | 29 |
| IL-10 Signaling^ | 1.62E+00 | 8 |  |  |  |  |
| Leptin Signaling in Obesity |  |  | 2.77E+00 | 11 |  |  |
