# Supplementary material for: Gene Expression Analysis in the Thalamus and Cerebrum of Horses Experimentally Infected with West Nile Virus
Source: PLoS One. 2011 Oct 4;6(10):e24371. doi: 10.1371/journal.pone.0024371 (PMC3186766; doi:10.1371/journal.pone.0024371)
Supplement: Table S7 — Functions for all analyses. The number of transcripts for significant functions for all analyses are listed. The * denotes functions involved with the nervous system (6) while the ∧ denotes functions involved with the immunological system (11). (DOCX) [file pone.0024371.s015.docx]

**Table S7. Functions for all analyses**

| **Function Category** | **Transcripts Exposure**  **Status** | **Transcripts Survival/Immune Status** | **Transcripts CNS Location** |
| --- | --- | --- | --- |
| Amino Acid Metabolism | 61 | 75 | 178 |
| ^Antigen Presentation | 2 |  | 8 |
| *Auditory and Vestibular System Development and Function |  | 7 |  |
| *Auditory Disease |  |  | 12 |
| *Behavior | 237 | 195 | 301 |
| Cancer | 648 | 918 | 893 |
| Carbohydrate Metabolism | 61 | 48 | 4 |
| Cardiovascular Disease | 744 | 505 | 618 |
| Cardiovascular System Development and Function | 61 | 37 | 44 |
| Cell Cycle | 174 | 239 | 390 |
| ^Cell Death | 1153 | 1082 | 719 |
| Cell Morphology | 214 | 230 | 326 |
| Cell Signaling | 107 | 60 | 164 |
| ^Cell-mediated Immune Response | 42 | 42 | 25 |
| Cell-To-Cell Signaling and Interaction | 361 | 453 | 455 |
| Cellular Assembly and Organization | 364 | 319 | 420 |
| Cellular Compromise | 32 | 11 | 8 |
| Cellular Development | 375 | 270 | 501 |
| Cellular Function and Maintenance | 140 | 63 | 104 |
| Cellular Growth and Proliferation | 806 | 721 | 424 |
| Cellular Movement | 546 | 671 | 699 |
| Connective Tissue Development and Function | 71 | 31 | 16 |
| Connective Tissue Disorders | 562 | 435 | 506 |
| Dermatological Diseases and Conditions | 20 | 11 | 18 |
| Developmental Disorder | 22 | 12 | 14 |
| DNA Replication, Recombination, and Repair | 46 | 16 | 35 |
| Drug Metabolism |  | 4 | 2 |
| Embryonic Development | 28 | 22 | 16 |
| Endocrine System Development and Function |  | 5 | 7 |
| Endocrine System Disorders | 518 | 502 | 566 |
| Gastrointestinal Disease | 404 | 345 | 328 |
| Gene Expression | 130 | 404 | 18 |
| Genetic Disorder | 1544 | 1269 | 1498 |
| Hair and Skin Development and Function | 2 | 2 | 4 |
| Hematological Disease | 12 | 22 | 13 |
| Hematological System Development and Function | 169 | 318 | 217 |
| Hematopoiesis | 40 | 81 | 50 |
| Hepatic System Development and Function | 3 | 3 |  |
| Hepatic System Disease | 13 | 13 |  |
| ^Humoral Immune Response | 20 | 18 | 29 |
| ^Hypersensitivity Response | 7 |  |  |
| ^Immune Cell Trafficking | 16 | 6 | 24 |
| ^Immunological Disease | 575 | 569 | 577 |
| ^Infection Mechanism | 12 | 21 | 3 |
| ^Infectious Disease | 164 | 54 | 58 |
| ^Inflammatory Disease | 965 | 798 | 824 |
| ^Inflammatory Response | 27 | 34 | 10 |
| Lipid Metabolism | 52 | 27 | 4 |
| Lymphoid Tissue Structure and Development | 7 | 2 |  |
| Metabolic Disease | 547 | 524 | 573 |
| Molecular Transport | 176 | 132 | 239 |
| *Nervous System Development and Function | 578 | 670 | 981 |
| *Neurological Disease | 1316 | 1210 | 1626 |
| Nucleic Acid Metabolism | 59 | 6 | 16 |
| Ophthalmic Disease | 2 | 2 | 18 |
| Organ Development | 36 | 14 | 64 |
| Organ Morphology | 24 | 9 | 20 |
| Organismal Development | 2 | 8 | 2 |
| Organismal Functions | 13 | 15 | 25 |
| Organismal Injury and Abnormalities | 11 | 16 | 61 |
| Organismal Survival | 155 | 140 | 126 |
| Post-Translational Modification | 2 | 200 | 238 |
| *Psychological Disorders | 195 | 164 | 322 |
| Renal and Urological Disease | 2 |  | 19 |
| Renal and Urological System Development and Function | 7 | 2 |  |
| Reproductive System Development and Function | 6 | 2 | 2 |
| Reproductive System Disease | 2 | 3 | 63 |
| Respiratory Disease | 2 | 53 | 6 |
| Respiratory System Development and Function |  | 3 |  |
| RNA Post-Transcriptional Modification | 2 | 4 |  |
| Skeletal and Muscular Disorders | 886 | 728 | 793 |
| Skeletal and Muscular System Development and Function | 92 | 39 | 14 |
| Small Molecule Biochemistry | 181 | 129 | 206 |
| Tissue Development | 102 | 245 | 234 |
| Tissue Morphology | 45 | 58 | 11 |
| Tumor Morphology |  | 5 | 18 |
| Vitamin and Mineral Metabolism | 24 | 14 | 64 |

Note: The number of transcripts for significant functions for all analyses are listed. The * denotes functions involved with the nervous system (6) while the ^ denotes functions involved with the immunological system (11).
