# Supplementary material for: Gene Expression Analysis in the Thalamus and Cerebrum of Horses Experimentally Infected with West Nile Virus
Source: PLoS One. 2011 Oct 4;6(10):e24371. doi: 10.1371/journal.pone.0024371 (PMC3186766; doi:10.1371/journal.pone.0024371)
Supplement: Table S8 — Transcripts for all analyses mapped to immunological CPs. This table shows the number of transcripts that mapped to each pathway for all analyses. The majority of the transcripts demonstrated a decrease in expression values. Transcripts were included if they demonstrated a fold change >1 or <−1. (DOCX) [file pone.0024371.s016.docx]

**Table S8. Transcripts for all analyses mapped to immunological CPs**

|  | Exposure | | Survival | | Location | |
| --- | --- | --- | --- | --- | --- | --- |
|  |  |  |  |  |  |  |
| 4-1BB Signaling in T Lymphocytes | 1 | 1 | 1 | 1 | 3 | 1 |
| Activation of IRF by Cytosolic Pattern Recognition Receptors | 1 | 3 | 0 | 5 |  |  |
| Acute Myeloid Leukemia Signaling |  |  |  |  | 4 | 1 |
| Acute Phase Response Signaling | 5 | 6 | 4 | 5 | 6 | 3 |
| Amyloid Processing |  |  | 3 | 1 |  |  |
| B Cell Activating Factor Signaling | 2 | 2 |  |  | 0 | 2 |
| B Cell Development |  |  |  |  | 0 | 3 |
| B Cell Receptor Signaling | 6 | 4 | 7 | 3 | 7 | 5 |
| Calcium-induced T Lymphocyte Apoptosis | 4 | 3 | 2 | 3 | 2 | 4 |
| Cardiac Hypertrophy Signaling |  |  | 15 | 4 | 14 | 6 |
| Caveolar-mediated \Endocytosis Signaling | 6 | 1 | 6 | 2 | 2 | 3 |
| CCR3 Signaling in Eosinophils | 7 | 2 | 8 | 2 | 8 | 4 |
| CCR5 Signaling in Macrophages | 3 | 2 | 4 | 3 | 4 | 4 |
| CD27 Signaling in Lymphocytes |  |  |  |  | 2 | 1 |
| CD28 Signaling in T Helper Cells | 6 | 5 | 2 | 4 | 3 | 5 |
| CD40 Signaling |  |  | 3 | 1 | 4 | 1 |
| Chemokine Signaling |  |  | 8 | 1 | 7 | 1 |
| Chronic Myeloid Leukemia Signaling |  |  |  |  | 4 | 2 |
| Clathrin-mediated Endocytosis Signaling | 13 | 1 | 7 | 1 | 9 | 3 |
| CNTF Signaling | 1 | 2 | 2 | 1 |  |  |
| CNTF Signaling |  |  |  |  |  |  |
| Complement System |  |  | 0 | 3 | 0 | 2 |
| CTLA4 Signaling in Cytotoxic T Lymphocytes | 4 | 3 | 3 | 3 | 5 | 5 |
| CXCR4 Signaling | 13 | 3 | 10 | 4 | 13 | 5 |
| Cytotoxic T Lymphocyte-mediated Apoptosis of Target Cells | 1 | 1 |  |  |  |  |
| Dendritic Cell Maturation |  |  | 3 | 6 | 4 | 5 |
| Fc Epsilon RI Signaling |  |  | 5 | 1 | 7 | 3 |
| Fcγ Receptor-mediated Phagocytosis in Macrophages and Monocytes |  |  | 4 | 2 | 6 | 4 |
| FcγRIIB Signaling in B Lymphocytes |  |  | 1 | 1 | 2 | 2 |
| FLT3 Signaling in Hematopoietic Progenitor Cells |  |  |  |  | 4 | 2 |
| fMLP Signaling in Neutrophils | 7 | 4 | 6 | 4 | 7 | 5 |
| GM-CSF Signaling | 3 | 2 | 4 | 1 | 6 | 2 |
| iCOS-iCOSL Signaling in T Helper Cells | 6 | 4 | 4 | 4 | 4 | 4 |
| IL-1 Signaling |  |  |  |  | 7 | 3 |
| IL-10 Signaling | 3 | 5 | 1 | 3 |  |  |
| IL-12 Signaling and Production in Macrophages |  |  | 3 | 3 | 6 | 4 |
| IL-15 Production | 0 | 4 | 1 | 3 | 1 | 4 |
| IL-15 Signaling |  |  | 2 | 1 | 4 | 3 |
| IL-17 Signaling |  |  |  |  | 4 | 2 |
| IL-2 Signaling |  |  | 3 | 0 | 5 | 2 |
| IL-22 Signaling | 0 | 3 | 1 | 2 | 1 | 2 |
| IL-3 Signaling | 4 | 2 | 4 | 2 | 6 | 4 |
| IL-4 Signaling |  |  |  |  | 2 | 3 |
| IL-6 Signaling | 4 | 2 | 3 | 2 |  |  |
| IL-8 Signaling | 11 | 3 | 9 | 7 | 9 | 8 |
| IL-9 Signaling | 1 | 2 | 1 | 2 | 2 | 3 |
| Interferon Signaling | 0 | 2 |  |  | 0 | 2 |
| Leukocyte Extravasation Signaling | 6 | 7 | 8 | 9 | 8 | 14 |
| LPS/IL-1 Mediated Inhibition of RXR Function | 7 | 7 |  |  |  |  |
| LPS-stimulated MAPK Signaling | 4 | 1 | 5 | 2 | 7 | 2 |
| Macropinocytosis Signaling | 4 | 1 | 3 | 3 | 4 | 5 |
| Mechanisms of Viral Exit from Host Cells |  |  | 3 | 2 | 5 | 1 |
| MIF Regulation of Innate Immunity |  |  | 2 | 1 | 2 | 0 |
| Natural Killer Cell Signaling |  |  | 4 | 4 | 7 | 5 |
| NF-κB Activation by Viruses |  |  | 4 | 3 | 5 | 6 |
| NF-κB Signaling | 8 | 3 | 4 | 4 |  |  |
| Oncostatin M Signaling | 0 | 3 | 1 | 1 | 2 | 2 |
| p38 MAPK Signaling | 3 | 3 | 3 | 2 |  |  |
| Primary Immunodeficiency Signaling | 1 | 4 | 1 | 5 | 0 | 5 |
| Production of Nitric Oxide and Reactive Oxygen Species in Macrophages | 9 | 4 | 6 | 6 | 9 | 7 |
| Regulation of IL-2 Expression in Activated and Anergic T Lymphocytes |  |  |  |  | 2 | 4 |
| Role of Macrophages, Fibroblasts and Endothelial Cells in Rheumatoid Arthritis |  |  | 11 | 8 | 9 | 8 |
| Role of NFAT in Regulation of the Immune Response | 10 | 7 | 7 | 8 | 7 | 8 |
| Role of Pattern Recognition Receptors in Recognition of Bacteria and Viruses | 1 | 5 | 2 | 5 | 3 | 4 |
| Role of PKR in Interferon Induction and Antiviral Response | 2 | 1 | 1 | 2 |  |  |
| Role of RIG1-like Receptors in Antiviral Innate Immunity | 1 | 2 | 0 | 4 |  |  |
| T Cell Receptor Signaling | 4 | 6 | 4 | 4 | 4 | 6 |
| T Helper Cell Differentiation | 1 | 2 |  |  |  |  |
| Toll-like Receptor Signaling | 3 | 2 |  |  |  |  |
| TREM1 Signaling |  |  |  |  | 2 | 1 |
| Virus Entry via Endocytic Pathways |  |  | 6 | 2 | 6 | 4 |
| Total | 176 | 130 | 215 | 166 | 266 | 210 |

This table shows the number of transcripts that mapped to each pathway for all analyses. The majority of the transcripts demonstrated a decrease in expression values. Transcripts were included if they demonstrated a fold change >1 or <-1.
