# Supplementary material for: Gene Expression Analysis in the Thalamus and Cerebrum of Horses Experimentally Infected with West Nile Virus
Source: PLoS One. 2011 Oct 4;6(10):e24371. doi: 10.1371/journal.pone.0024371 (PMC3186766; doi:10.1371/journal.pone.0024371)
Supplement: Table S9 — Specific clinical signs of horses by group analyzed by CNS microarray. Horses were graded as no increase in clinical signs, a moderate to mild increase in clinical signs, and a severe increase in clinical signs for all studies. Clinical signs observed included changes in mentation, paresis, ataxia, and muscle fasciculations. The nonvaccinated group exposed to West Nile virus demonstrated the most severe increase in severity of clinical signs, while the vaccinated/exposed group and normal controls did not show any changes in clinical signs. (DOCX) [file pone.0024371.s017.docx]

**Table S9. Specific clinical signs of horses by group analyzed by CNS microarray**

| **Treatment** | **Clinical Parameter** | **No Increase (%)** | **Mild to Moderate (%)** | **Severe (%)** |
| --- | --- | --- | --- | --- |
| **Infected Vaccinated Horses (6)** | **Change or Severity of Mentation** | 6(100) | 0(0) | 0(0) |
|  | **Paresis** | 6(100) | 0(0) | 0(0) |
|  | **Ataxia** | 6(100) | 0(0) | 0(0) |
|  | **Fasciculations** | 6(100) | 0(0) | 0(0) |
| **Infected**  **Nonvaccinated Horses (6)** | **Change or Severity of Mentation** | 2(33.3) | 3(50) | 1(16.7) |
|  | **Paresis** | 1(16.7) | 3(50) | 2(33.3) |
|  | **Ataxia** | 0(0) | 3(50) | 3(50) |
|  | **Fasciculations** | 0(0) | 1(16.7) | 5(83.3) |
| **Noninfected Horses (6)** | **Change or Severity of Mentation** | 0(0) | 0(0) | 0(0) |
|  | **Paresis** | 0(0) | 0(0) | 0(0) |
|  | **Ataxia** | 0(0) | 0(0) | 0(0) |
|  | **Fasciculations** | 0(0) | 0(0) | 0(0) |
